# From incubation to release: Hand-rearing as a tool for the conservation of the endangered African penguin

**DOI:** 10.1371/journal.pone.0205126

**Published:** 2018-11-07

**Authors:** Romy Klusener, Renata Hurtado, Nola J. Parsons, Ralph Eric Thijl Vanstreels, Nicola Stander, Stephen van der Spuy, Katrin Ludynia

**Affiliations:** 1 Southern African Foundation for the Conservation of Coastal Birds, Cape Town, Western Cape, South Africa; 2 Institute of Research and Rehabilitation of Marine Animals, Cariacica, Espírito Santo, Brazil; 3 Marine Apex Predator Research Unit, Institute for Coastal and Marine Research, Nelson Mandela University, Port Elizabeth, Eastern Cape, South Africa; 4 DST-NRF Centre of Excellence at the FitzPatrick Institute for African Ornithology, Department of Zoology, Nelson Mandela University, Port Elizabeth, Eastern Cape, South Africa; 5 Department of Biological Sciences, University of Cape Town, Cape Town, Western Cape, South Africa; University of Minnesota, UNITED STATES

## Abstract

The African penguin (*Spheniscus demersus*) population is estimated at 25,000 breeding pairs, approximately 5% of that at the start of the 20th century, and the species is currently classified as Endangered. In the last two decades, the hand-rearing of penguin chicks that were abandoned by their parents due to oil spills or other circumstances has become a valuable conservation tool to limit mortality and to bolster the population at specific colonies. We summarize and evaluate the techniques employed by the Southern African Foundation for the Conservation of Coastal Birds (SANCCOB) to incubate and hand-rear African penguin eggs and chicks. From 2012 to 2016, a total of 694 eggs and 2819 chicks were received by SANCCOB’s Chick Rearing Unit. It was estimated that 13% of the eggs were infertile, and 81% of the fertile eggs hatched successfully. The overall release rate for chicks was 77%, with a higher release rate for chicks that were pre-emptively removed (93%) followed by chicks that had been abandoned by their parents (78%), chicks admitted due to avian pox lesions (61%), chicks that hatched from artificially-incubated eggs (57%), and chicks admitted due to injuries or deformities (25%). Rescuing and hand-rearing eggs and chicks has been a successful strategy for African penguins, and might be also applicable for the conservation of other threatened seabird species whose population are critically low or during natural or anthropogenic events that could have disastrous population impacts (e.g. oil spills, disease outbreaks, catastrophic weather events, strong El Niño years, etc.).

## Introduction

The African penguin (*Spheniscus demersus*) is endemic to the greater Agulhas-Benguela upwelling ecosystem of southern Africa. The present population is approximately 5% of that at the start of the 20th century, when it was estimated at over 1.45 million adults [1]. Historically, African penguin eggs were harvested in large numbers, causing large-scale population declines [2]. By the late 1970s the population had declined to 220,000 adults, due to unsustainable egg harvesting and oil pollution [1,3]. By the 1990s only 179,000 adults remained due to massive disturbance and habitat alteration caused by the collection of guano [1], and the species was classified as Near Threatened [4]. The species’ population continued declining in the 21^st^ century due to changes in the abundance, distribution and quality of food, and between 2001 and 2009 the number of African penguins further declined by 60%, reaching a global population of 26,000 breeding pairs (*c*. 83,200 individuals) and was reclassified as Vulnerable [4,5]. The last assessment on the global population of African penguins was conducted in 2015 and estimated the global population at 25,000 breeding pairs (*c*. 80,000 individuals), and the species was classified as Endangered in 2010 [4].

At present, the population decline is attributed to a decreased availability of food, resulting from shifts in the distribution of prey species, competition with commercial fisheries and environmental fluctuations [5,6]. Given the large decrease in the past centuries and the undergoing threats to African penguins there is considerable concern on the long-term viability of the species in the wild [7]. Efforts for the conservation of the African penguin have focused on strategies to manage fisheries and improve breeding success, such as providing artificial nests, rehabilitation of oiled and injured adults, and hand-rearing of abandoned chicks [7–10].

In the last two decades, the hand-rearing of penguin chicks that were abandoned by their parents due to oil spills or other circumstances (e.g. abandonment due to onset of moult, catastrophic weather events) has become a valuable conservation tool to limit mortality and to bolster the population at specific colonies [7,9–11]. Past studies have shown that hand-reared chicks have survival and recruitment rates comparable to those of naturally-reared cohorts, and reproduce successfully after they reach sexual maturity [9,10]. In this study, we summarize and evaluate the techniques that have been employed by the Southern African Foundation for the Conservation of Coastal Birds (SANCCOB) to incubate and hand-rear African penguin eggs and chicks since the implementation of their specialized Chick Rearing Unit in November 2011.

## Materials and methods

**Fig 1:**
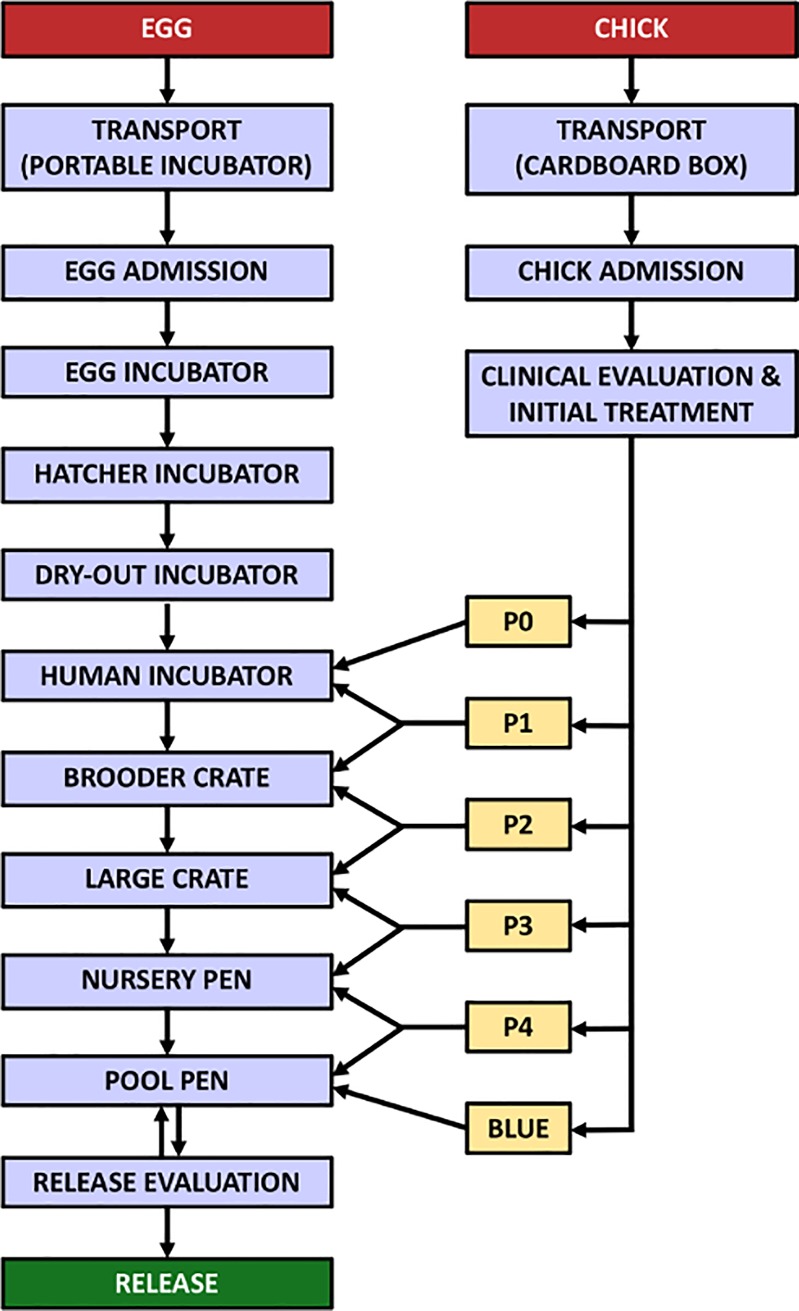
Overview of the stages of the incubation and hand-rearing of African penguin eggs and chicks at SANCCOB.

**Fig 2:**
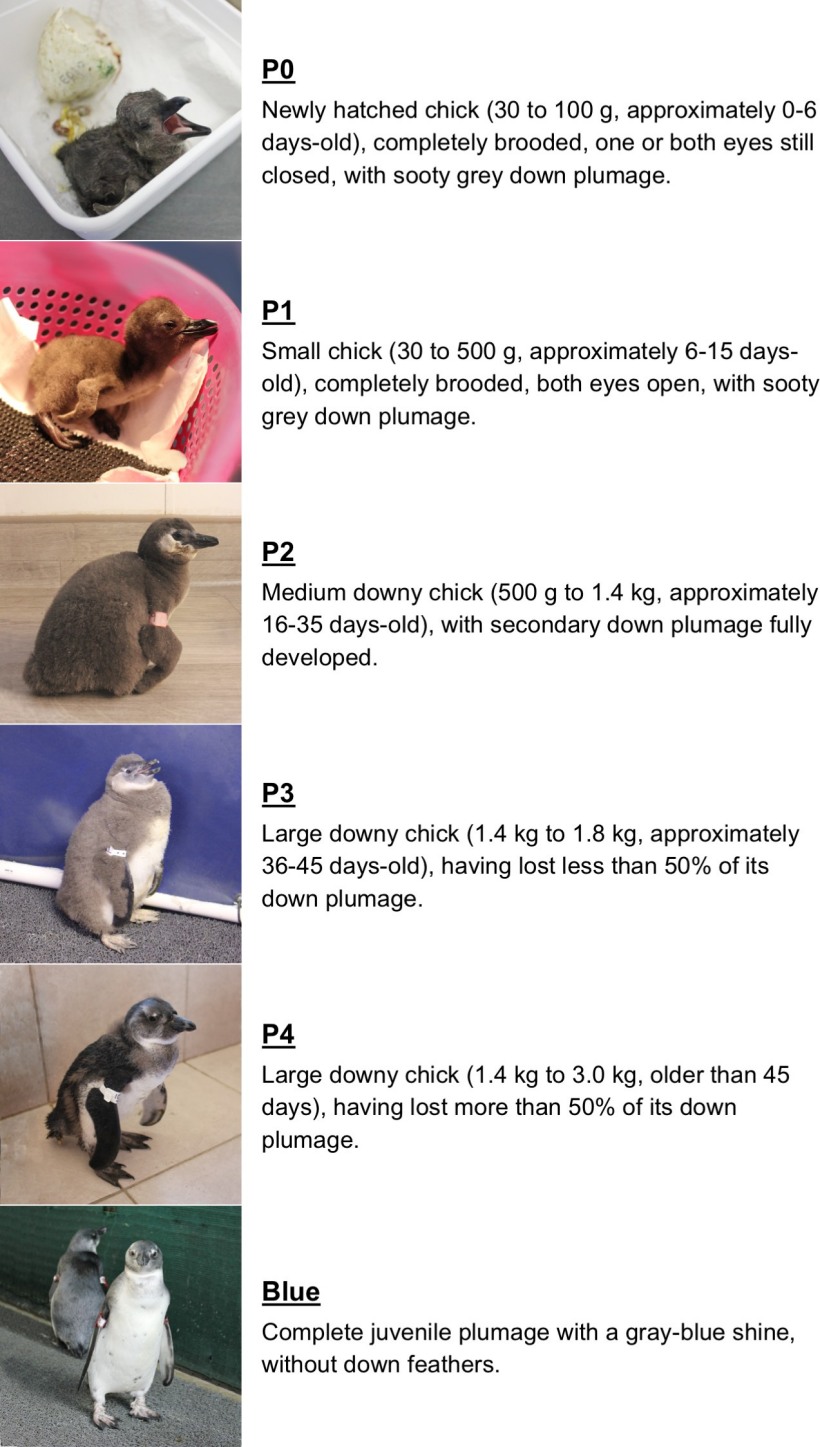
Classification of African penguin chick developmental stages. This classification scheme was based on Barham et al. [9] and Sherley et al. [10].

Incubation and hand-rearing procedures are conceptually outlined in Fig 1. Chicks were classified into six stages of development based on their size, plumage and other external characteristics (Fig 2) based on Barham et al. [9] and Sherley et al. [10]. The rehabilitation protocols were based on incubation and hand-rearing protocols developed for other penguin species [12–14], and were empirically adapted based on the breeding biology of the African penguin [3,15–17] and on the continuous re-evaluation of the rehabilitation results. A detailed description of the African penguin incubation and hand-rearing procedures is provided in S1 File.

### Removal and transportation of eggs and chicks

Between January 2012 and December 2016, African penguin eggs and chicks were occasionally removed from breeding colonies in the Western Cape province of South Africa: Jutten Island (33°04'59"S 17°57'19"E), Dassen Island (33°25'24"S 18°05'10"E), Robben Island (33°48'21"S 18°22'06"E), Boulders (34°11'50"S 18°27'04"E), Stony Point (34°22'26"S 18°53'41"E) and Dyer Island (34°40'56"S 19°25’03"E).

The reason for admission was classified into five groups (Table 1): egg, abandonment, pre-emptive removal, avian pox, and injury or deformity. Eggs were removed when they considered to have been abandoned (when rangers noted that it was not being incubated or attended to by an adult) or were pre-emptively removed when colony staff considered they were in nests in high risk areas (e.g. potential dog predation, civil construction, excessive proximity to roads) or in private property and the owners requested their removal. P0 and P1 chicks were considered to have been abandoned when they were not accompanied by an adult. P2 to P4 chicks and Blues were considered to have been abandoned when the rangers noted that they were not being attended for an extended period of time or when they showed signs of malnutrition, dehydration, hypothermia or excessive pecking wounds to the head or back. Chicks were pre-emptively removed from their nests when colony staff considered they were in high risk areas, when nests were potentially at risk of flooding or when they were on private property and the owners requested their removal. The “avian pox” category comprised chicks that were removed because they presented wart-like lesions or tumours consistent with cutaneous pox [18]. The “injury or deformity” category comprised chicks that suffered trauma (e.g. dog or predator attack) or had deformities of known or unknown aetiology (e.g. skewed beak).

**Table 1:** Numbers of African penguin eggs and chicks admitted for hand-rearing from different breeding colonies at the SANCCOB Cape Town facility between 2012 and 2016.

	Jutten Island	Dassen Island	Robben Island	Boulders	Stony Point	Dyer Island	Total
**Reason for admittance**							
Egg	-	-	4	458	27	-	489
Abandonment	3	47	107	129	1835	167	2288
Pre-emptive removal	-	-	-	480	-	-	480
Avian pox	-	-	-	-	23	-	23
Injury or deformity	-	2	4	13	9	-	28
**Developmental stage**							
Egg	-	-	4	458	27	-	489
P0 chick	-	-	3	17	10	-	30
P1 chick	-	1	4	76	45	1	127
P2 chick	-	6	27	220	429	22	704
P3 chick	1	10	15	120	501	50	697
P4 chick	2	28	50	158	737	66	1041
Blue	-	4	12	31	145	28	220
**Total**	3	49	115	1080	1894	167	3308

After collection, eggs were placed horizontally in foam-lined cases designed for egg transportation and set in a portable incubator at 30°C. Chicks were accommodated (max. of three individuals with similar sizes) in aerated cardboard boxes with towels on the bottom. Eggs and chicks were transported on the same day and then admitted to the Chick Rearing Unit (CRU) at SANCCOB’s Cape Town facility (33°50’02”S 18°29’29”E).

### Egg incubation

On admission, eggs received an identification number marked with pencil on the shell, were candled (to check viability and estimate stage of development), weighed (precision 0.01 g), measured (length and breadth), and then placed in a pre-heated incubator set at 36.5°C and 45% humidity (egg incubator stage). The incubator was set to continually rotate eggs width-ways (complete rotation every two hours), and eggs were manually rotated 180° lengthways once every 24 hours.

Eggs were weighed daily and candled weekly. Once the eggs externally pipped (i.e. when the shell was first cracked by the hatching chick), they were placed in a plastic container with soft paper towels and moved to an incubator set at 36.5°C with ~60% humidity without manual or automatic rotation (hatcher incubator stage). If a chick did not hatch on its own 48 to 50 hours after external pipping was first noticed, hatching was assisted.

After a chick hatched (completely detached from the egg), it was checked for abnormalities, weighed (precision 0.01 g), placed in a container lined with clean soft paper towels, and transferred to a pre-heated incubator set at 36.5°C and 45% humidity for 12 hours to dry out its fluff (dry-out incubator stage). To compensate for the lack of vertical transmission of gut flora, probiotics were orally administered (see S1 File). Once the chick was dry, it was placed in individual containers lined with clean soft paper towels, and transferred to a human baby incubator (human incubator stage), entering the hand-rearing process.

Eggs were discarded if there was any oozing of the egg or bad smell present, if their yolk showed no development after 14 days of incubation, if an egg did not hatch after 2 weeks past the estimated hatching date (especially if the egg had been incubated since the yolk or small embryo stage), or if after 7–13 days the egg presented one of the following characteristics: a dark black spot was seen instead of embryo without surrounding veins, there was a solid dark red ring around a dead embryo with no vein network, an air bubble was seen floating in the dark part of the egg (often near to the air sac), or the dark part of the egg appeared liquid and not a solid embryo. Whenever feasible, discarded eggs were examined to determine if an embryo was present or not, and to establish the embryonic stage of development: early (very small embryo, approximately 2 cm, large head with large eyes, poorly developed limbs), mid (head relatively well-proportioned to the body, limbs well developed, no fluff), late (fully developed, fluff present, about to hatch), or deceased during hatching.

### Chick admission

During admission, all chicks received an individual identification number and temporary tags (leg tags for P0 and P1 chicks, flipper tags for P2-P4 chicks and Blues). Chicks were also physically examined, weighed, subjected to respiratory auscultation and their hydration was evaluated. Blood samples were collected from P2 or older chicks with a mass greater than 1 kg. Depending on their clinical condition, chicks received oral electrolyte hydration or parenteral fluid therapy (detailed in S1 File). To reduce ectoparasite load, upon admission chicks (P2 or older) had their plumage sprinkled with insecticide powder (carbaryl 50 g/kg) on the back and abdomen. For P0 and P1 with ectoparasites, an insecticide solution (fipronil 2.5 g/L) was sprayed onto a swab and then dabbed down the back of the chick. Initial treatment was administered according to each bird’s mass and hydration status. Chicks weighing 500 g or more received an initial dose of deworming drugs (ivermectin 0.2 mg/kg and praziquantel 7.5 mg/kg, same dose repeated after 14 days). Depending on its developmental stage, and also taking into account their clinical condition, body mass and behaviour, each chick was assigned to a stage of the hand-rearing process (Fig 1).

### Chick hand-rearing

To limit disease transmission from older to younger chicks, hand-rearing was conducted in three separate buildings. The CRU receives eggs, and chicks under 1.5 kg (P0 to early P3 stages), whereas the Nursery Unit receives chicks above 1.5 kg (P3 and P4 stages). Finally, when chicks have lost their downy plumage (Blue stage) they are moved to a pool pen at the general rehabilitation facilities (where adult penguins and other seabirds are routinely rehabilitated). Because hatchlings and abandoned chicks are usually immune-compromised, both the CRU and Nursery Unit have strict hygiene protocols (detailed in S1 File) and access is restricted to essential personnel.

P0 chicks were housed individually in small plastic containers with clean soft paper towels and transferred to an incubator set at 33°C and 45% humidity for 5 days (human incubator stage). From day 6 to day 8, the temperature of the incubator was lowered to 30°C. After day 8 (or when chicks reached 100 g), the chick (now P1 stage) was transferred to a crate kept under infrared heat lamps (brooder crate stage). When chicks reached 500 g and were in good clinical condition, they were moved to larger crates with one other chick in order to allow socialization (large crate stage). Large crates were kept in a well-ventilated room (18–21°C) and were taken outdoors on days with good weather.

Chicks under 1.5 kg were weighed every day (precision 0.01 g) before their first feed to monitor its development and calculate the quantity of their diet for the day. The feeding regimen is detailed in S1 File. Briefly, chicks received oral fluids and were fed several times a day for 10% of their body mass at each feed. Each meal consisted of fish formula (blended fish with vitamin and mineral supplements and probiotics), fish fillets, fish tails or whole fish depending on their developmental stage and body mass. When chicks reached 1.5 kg, they were transferred to the Nursery Unit (nursery pen stage), where they were housed in an external pen during the day and an internal pen during the night (or during rainy or excessively cold days). When most of the downy plumage had been lost (some P4 and all Blue stages), penguins were transferred to the pool pens for pre-release conditioning.

### Pre-release conditioning

Blue-stage penguins were permanently housed outdoors with pool access (pool pen stage), in large groups (occasionally sharing the pen with juvenile and adult African penguins). During the following weeks, the birds were subjected to a progressive swimming schedule, depending on their feather grading and behaviour (S1 File). Feather grading was performed weekly by force-swimming the penguins for 10–20 min then verifying the waterproofing of their plumage (routine grading) in order to evaluate their progress and group them with other penguins in a similar stage of the conditioning process.

When the Blue-stage penguins were approved for other release criteria (apparently healthy, good body condition, normal behaviour and swimming, not imprinted on humans), a more comprehensive feather grading was conducted after force-swimming them for 60 min (pre-release grading) to ensure that only individuals with fully waterproof plumage were released. Penguins that met the release criteria were microchipped and then released at the Stony Point or Boulders colonies, during the morning of days where good weather had been forecast (swell no more than 2.5–3 m with a gentle breeze under 20 km/h).

### Permits, animal welfare, preventive medicine and veterinary care

Incubation and hand-rearing procedures were conducted with national permits from the Department of Environmental Affairs under the Seabirds and Seals Protection Act (Act 46 of 1973) (permits OC/CBC/2013/016, OC/OCS/013, OCS/2016/04) and the Threatened or Protected Species permit system (permit 03314, registration 29151) and with provincial permits from CapeNature (permit 0023-AAA004a00121). These permits authorised SANCCOB to capture, collect, hold, transport, care for and rear oiled, sick, injured and orphaned birds, including their chicks for conservation, rehabilitation and humane purposes. The use of the data for this study was also approved by SANCCOB’s internal research ethics committee.

Access to the incubation and hand-rearing facilities was restricted to trained staff and interns, and all procedures were done under the supervision of onsite veterinarians and a full-time staff with extensive training on animal husbandry and welfare. This allowed for the prompt detection and correction of any problems relating to stress or animal welfare. Whenever possible, chicks were placed with other chicks to mimic their natural environment, and environmental enrichment items (plush toys or kennels) were provided throughout the hand-rearing process. Facilities were designed to minimise acoustic, olfactory and visual stress, and all efforts were made to keep handling time and other stressors to a minimum. Euthanasia (inhalational general anaesthesia followed by intra-venous injection of euthanasia agent) was employed by the veterinary staff to interrupt suffering in cases where chicks were in pain (e.g. admitted due to severe trauma) or where there was no perspective of clinical improvement and release to the wild (e.g. severe congenital deformities).

Routine veterinary checks were conducted on a weekly basis. A small volume of blood (*c*. 0.1 mL) was obtained weekly from penguins weighing over 1.5 kg, to evaluate packed cell volume, buffy coat, total plasma protein and blood smears to diagnose blood parasites and to quantify erythrocytic regeneration and leukocytes [19]. Faecal samples were collected and fresh smears were examined whenever a penguin presented loss of appetite, slow weight gain or other signs of gastrointestinal problems. A special nutritional regimen was administered for penguins at P4 or Blue stage admitted with emaciation (body mass equal to or lower than 75% of the normal body mass for their size) (see S1 File). To prevent exposure to flies and mosquitoes, all SANCCOB facilities were netted, pyrethroid-based pest repellent diffusers were routinely used, and insecticides (diethyl-meta-toluamide 35% roll-on stick) were routinely applied to the birds’ heads.

### Data analysis

Information on the location of removal and reason for admittance of eggs and penguin chicks admitted was compiled for descriptive analysis. Daily weighing data from a subset of 60 eggs that hatched successfully (1311 data points) was used to evaluate the following variables: egg mass upon admission, egg mass on the day preceding external pipping, chick mass upon hatching and daily progression of egg mass during incubation. For the same subset of eggs, the interval (in days) from admission to external pipping and hatching was also evaluated. Egg volume (V) was calculated from the length (L) and breadth (B) using the equation V = 5.5101 + 0.4837LB^2^ [20], and egg density was calculated by dividing an egg’s mass by its volume. Daily weighing data from a subset of 50 chicks that survived to be released (4004 data points) was used to evaluate the growth curve.

Chick outcome was classified as release or death, and the latter category comprised both spontaneous death and euthanasia. Hatching rate was calculated as the number of eggs that hatched successfully divided by the number of eggs admitted. Release rate was calculated as the number of chicks released divided by the number of chicks admitted. Linear and non-linear regression analysis as implemented in Past 3.16 [21] was used to evaluate which equation would best fit the temporal progression of the following parameters: number of eggs admitted per month, hatching rate per month, chick body mass per day, and chick daily relative mass change per day. The R-squared value and Akaike’s Information Criterion were used to determine which model best fit the data, and the data was subdivided using empirical thresholds when it was clear that a single model could not adequately fit the data. The raw datasets analysed in this study are provided in S2 File.

## Results

**Fig 3:**
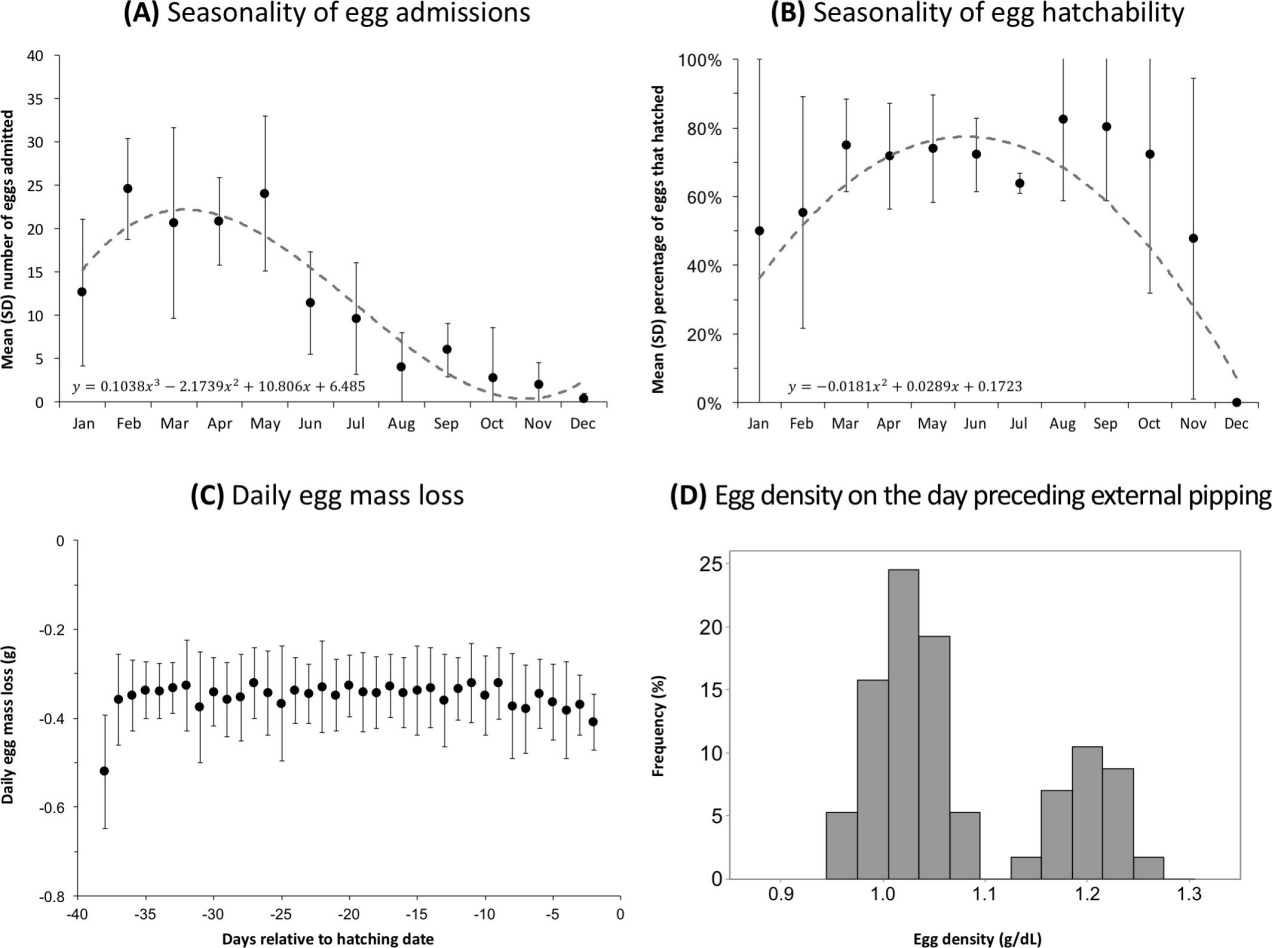
**Monthly distribution of the number of eggs admitted (A) and hatching rate (B), daily changes in egg mass loss (C), and histogram of egg density on the day preceding external pipping (D).** Graphs (A) and (B) are based on all eggs admitted to the SANCCOB Chick Rearing Unit between 2012 and 2016 (N = 694). Graphs (C) and (D) are based on a subset of eggs that hatched successfully (N = 60).

A total of 694 eggs were admitted between 2012 and 2016, and 489 of those eggs (70%) hatched successfully. The number of eggs admitted was highest from February to May (Fig 3A) and the hatching rate was highest for eggs admitted in fall (April to June) (Fig 3B). Of the eggs that did not hatch successfully and were necropsied, 110 eggs (57%) contained a dead embryo, 83 eggs (43%) did not contain a visible embryo and were presumed to be infertile; no necropsy was conducted for 12 eggs. The stage of embryonic development upon death was determined for 72 embryonated eggs: 22 eggs (31%) were in an early stage, 12 eggs (17%) were in an intermediate stage, 26 eggs (36%) were in a late stage, and 12 chicks (17%) died during hatching. If it is presumed that 43% of the eggs that were not necropsied were infertile, it may be estimated that 87% of the eggs were fertile (i.e. 606 fertile eggs, 88 infertile eggs), and therefore 81% of the fertile eggs hatched successfully.

**Fig 4:**
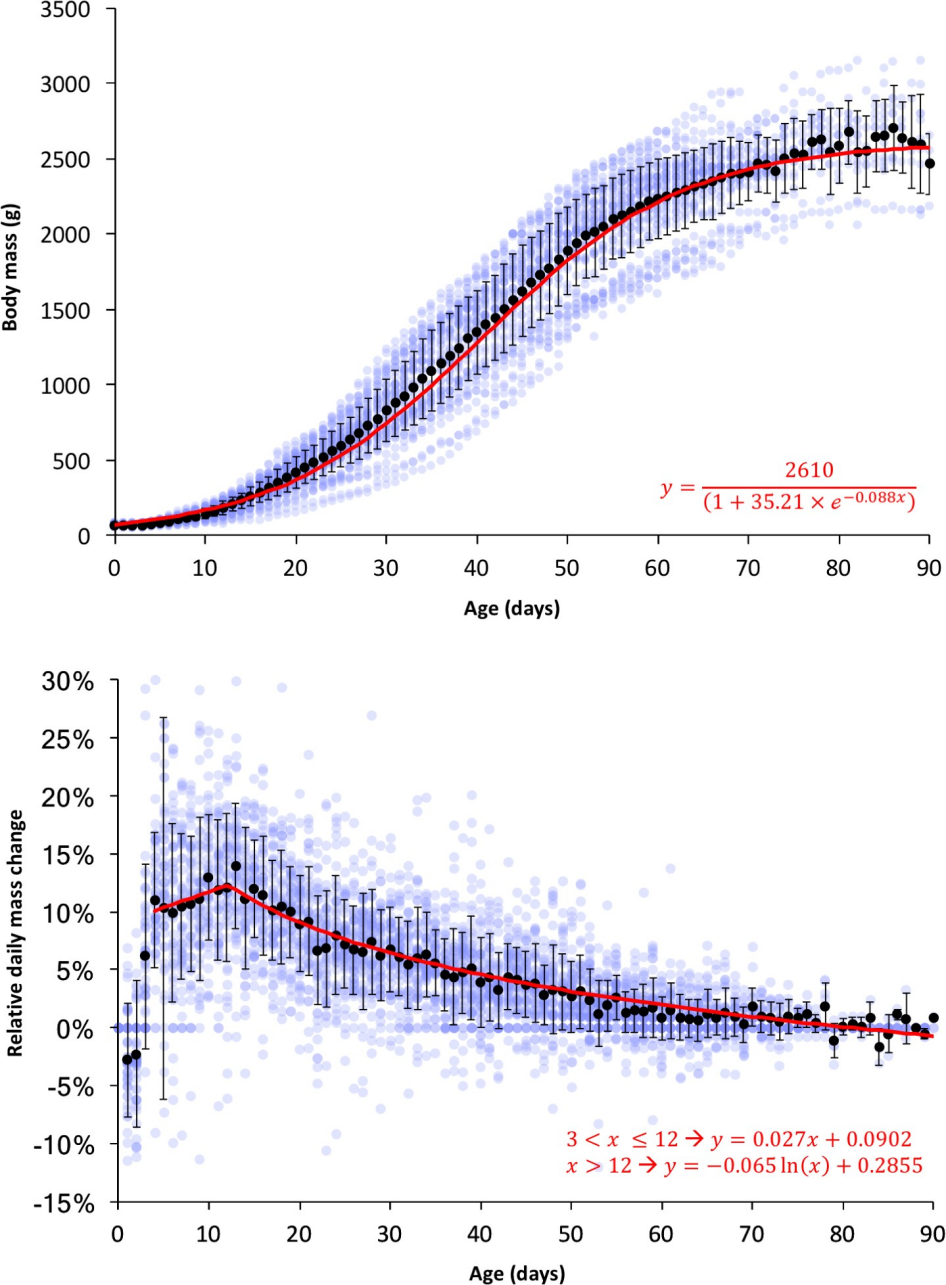
**Growth curve (top) and daily relative mass change (bottom) of African penguin chicks.** Both graphs are based on a subset of chicks that hatched at the SANCCOB Chick Rearing Unit and survived to be released (N = 50). Blue dots represent data points, black circles with vertical bars represent mean values and the standard deviation, and red lines represent mathematical models (equations are shown).

Table 2 provides a summary of the different quantitative parameters relating to egg incubation and hatching, based on a subset of 60 eggs that hatched successfully. Fig 3A and 3B summarize the seasonal distribution of the number of eggs admitted and their hatchability, respectively. Regardless of their mass upon admission, eggs lost on average 0.35 ± 0.09 grams per day (mean ± SD), and this rate was stable throughout the incubation period (Fig 3C). Egg density also changed at a stable rate throughout the incubation, with an average decrease in the density of 0.004 ± 0.001 g/mL per day. The distribution of egg density was bimodal, with one group of eggs reach presenting a density of 0.96 to 1.07 on the day preceding external pip, and another group of eggs presenting a density of 1.14 to 1.27 on the day preceding external pip (Fig 3D). Fig 4A shows the daily progression of body mass of a subset of chicks hatched at SANCCOB that survived to be released, and Fig 4B shows the daily relative mass change for the same individuals.

**Table 2:** Descriptive statistics of quantitative parameters of the incubation of African penguin eggs (N = 60).

Parameter	Mean	S.D.	Minimum	Maximum
Egg length (cm)	68.99	3.55	60.90	76.40
Egg breadth (cm)	51.37	2.71	44.20	59.30
Egg volume (mL)	87.60	10.37	62.37	110.01
Egg mass upon admission (g)	98.30	11.35	71.42	130.67
Egg mass on the day preceding external pip (g)	92.62	10.01	68.05	117.30
Egg density upon on the day preceding external pip (g/mL)	1.075	0.088	0.960	1.270
Chick mass upon hatching (g)	72.07	9.14	52.00	92.20
Chick-to-egg mass ratio upon hatching (%)	77.79	2.23	69.99	82.17
Average daily absolute egg mass change (g)	-0.35	0.09	-0.94	0.00
Average daily relative egg mass change (%)	-0.36	0.11	-1.00	0.00
Average daily relative egg density change (g/mL)	-0.004	0.001	-0.017	0.00
Interval from admission to external pip (days)	22.2	10.5	1	37
Interval from admission to hatching (days)	24.1	10.5	3	39
Interval from external pip to hatching (days)	1.8	0.4	0	2

**Fig 5:**
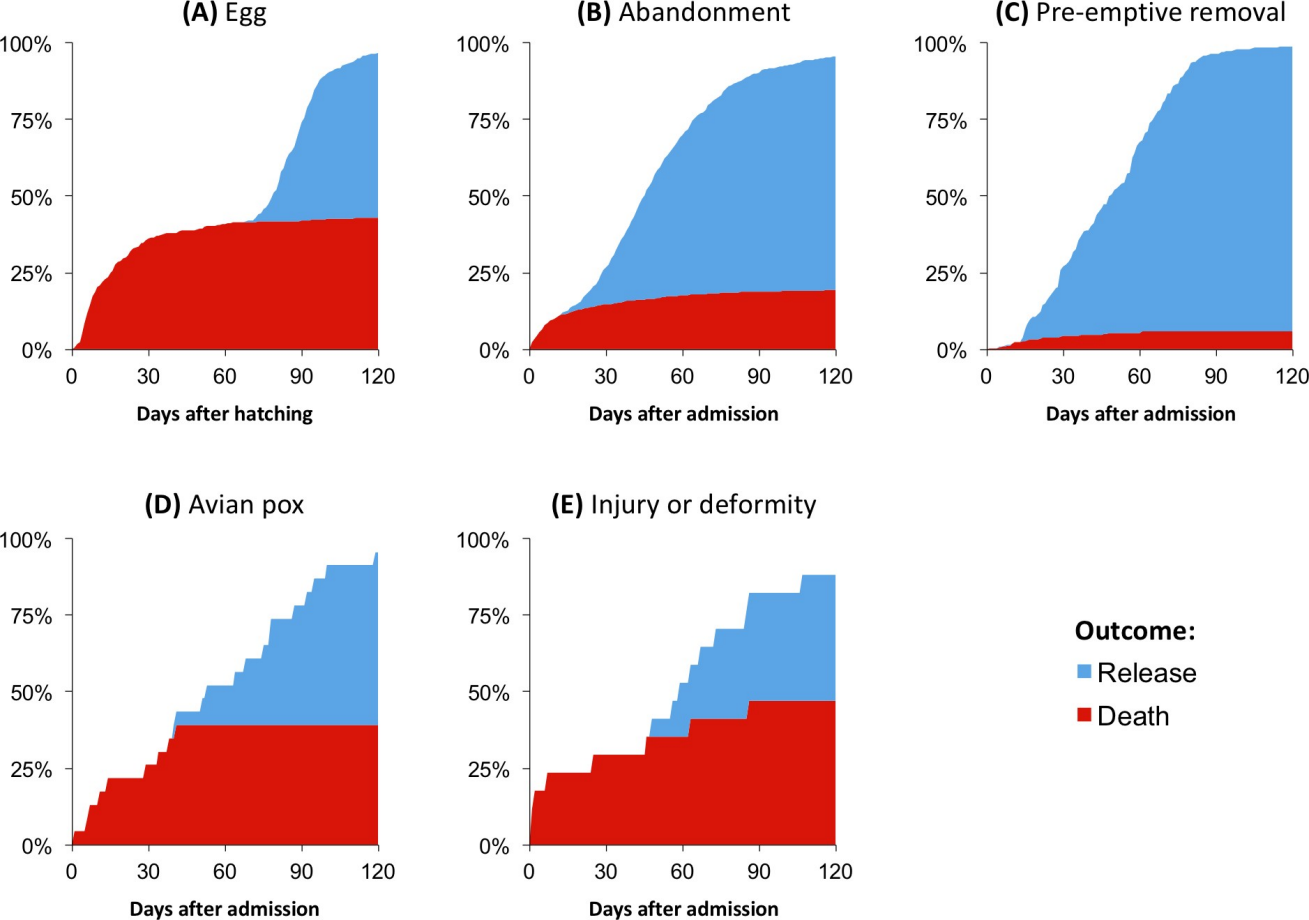
Cumulative distribution of the outcomes of hand-reared African penguin chicks during the first 120 days following hatching or admission. Each graph represents individuals with a different reason for admission: (A) egg (N = 489), (B) abandonment (N = 2288), (C) pre-emptive removal (N = 480), (D) avian pox (N = 23), and (E) injury or deformity (N = 28).

In addition to the 489 chicks that were hatched from eggs admitted to SANCCOB, an additional 2819 chicks in different developmental stages were admitted for hand-rearing between 2012 and 2016 (Table 1). The overall release rate was 76.7% (2538 chicks released out of 3308 chicks hatched or admitted), with considerable variation depending on the developmental stage upon admission and the reason for admittance (Table 3). For eggs, the release rate was 39.9% (277 chicks released out of 694 eggs admitted); if it is considered that 43% of the failed eggs that were necropsied were found to be infertile, it may be estimated that the release rate was 45.7% for fertile eggs (277 chicks out of 606 eggs). The outcomes (death or release) were unevenly distributed depending on the reason for admittance and developmental stage (Fig 5), with nearly all the chicks having reached their outcome within 120 days from hatching or admission.

**Table 3:** Release rate of hand-reared African penguin chicks in relation to the reason of admittance and the developmental stage upon admission. Results are presented as “individuals released/individuals admitted (% release rate)”.

Admitted as	Egg	Abandonment	Injury or deformity	Avian pox	Pre-emptive removal	Total
Egg	277/489 (57%)	-	-	-	-	277/489 (57%)
P0 chick	-	5/15 (33%)	-	-	4/15 (27%)	9/30 (30%)
P1 chick	-	38/57 (67%)	1/1 (100%)	-	58/69 (84%)	97/127 (76%)
P2 chick	-	377/489 (77%)	3/11 (27%)	14/22 (64%)	179/182 (98%)	573/704 (81%)
P3 chick	-	434/592 (73%)	0/5 (0%)	-	98/100 (98%)	532/697 (76%)
P4 chick	-	757/933 (81%)	3/9 (33%)	-	96/99 (97%)	856/1041 (82%)
Blue	-	181/202 (90%)	0/2 (0%)	0/1 (0%)	13/15 (87%)	194/220 (88%)
Total	277/489 (57%)	1792/2288 (78%)	7/28 (25%)	14/23 (61%)	448/480 (93%)	2538/3308 (77%)

The release rate for all chicks was lowest in 2012 (59.0%) and 2013 (70.6%), and was higher in 2014 (82.1%), 2015 (86.1%) and 2016 (82.7%). For chicks hatched at SANCCOB, the release rate increased even more markedly over the years: 2012 (31.3%), 2013 (22.6%), 2014 (58.5%), 2015 (75.5%), and 2016 (87.1%). For 2016 (N = 613), the release rate was highest for the pre-emptive removal (97.1%), followed by chicks hatched at SANCCOB (87.1%), abandonment chicks (80.7%), and injury or deformity (40%).

## Discussion

Rehabilitation is valuable strategy for the mitigation of human impacts on the African penguin population, as exemplified by the large-scale rescue and rehabilitation operations conducted in response to the *Apollo Sea* (1992) and *Treasure* (2000) oil spills [1,9,22–24]. In recent years, SANCCOB’s rehabilitation efforts have been redirected to address an emerging challenge, with the recurring abandonment of eggs and chicks and the resulting poor breeding success. The African Penguin Chick Bolstering Project was established in 2006 in an attempt to mitigate the impacts of the abandonment of eggs and chicks, aiming to slow the rate of population decline [7,11].

Depending on the breeding colony and year, the hatching rate of naturally-incubated African penguin eggs can range from 35 to 85% [3,16,25,26]. The hatching rate of 70% obtained in artificially-incubated eggs in this study may therefore be considered satisfactory, especially if it is considered that this rate rises to 81% when only fertile eggs are considered. We estimate that 13% of the eggs admitted to SANCCOB were infertile, which is comparable to the 10–18% infertility rate in wild yellow-eyed penguins (*Megadyptes antipodes*) [27]. Although African penguins can lay eggs year-round, the seasonal distribution in the number of eggs admitted is consistent with the peaks of egg laying in the Western Cape [28,29]. On the other hand, the seasonal differences in egg hatchability may be related to the fact that eggs left unattended during hot months are more likely to overheat [30,31].

Compared to other penguin species, the eggs of African penguins have limited dimorphism; the mean mass upon laying is 106.8 g for A-eggs (range: 75–132 g) and 104.8 g for B-eggs (range: 83–129 g) [32]. Based on the formula provided by Rahn and Ar [33], eggs of such mass are expected to lose approximately 0.47 g per day during incubation (range: 0.37–0.56 g); the mean loss of 0.35 g per day as observed in this study is therefore slightly lower than would be theoretically expected. The rate of egg mass loss was constant during the entire incubation and was not influenced by egg mass, which is consistent with the interpretation that egg mass loss is a passive process due to evaporation, which in turn is determined by incubation parameters (temperature and humidity). Future studies on naturally-incubated African penguin eggs would therefore be valuable in order to determine what is the natural daily egg mass loss and how the artificial incubation parameters (temperature and humidity) could be optimized in order to maximise hatching rate.

The equation we used to estimate egg volume does not take into account the slight differences in shape between A- and B-eggs, and thus will tend to overestimate the volume of A-eggs and underestimate the volume of B-eggs [20]. For this reason, we believe that the bimodal distribution observed in egg density on the day preceding external pipping (Fig 3D) should not be interpreted as indicative of actual differences in egg density, but instead probably reflects an inaccurate estimation of egg volume due to shape dimorphism. Interestingly, however, these results suggest that egg density may be useful in order to differentiate A- and B-eggs when the laying order is not known; further studies using data from eggs for which the laying order is known would therefore be valuable to confirm this.

Because of their lower body mass-to-surface ratio and developing physiological and immunological functions, chicks in earlier stages of development can be expected to be more vulnerable to health challenges than those in later stages. This is reflected by the lower survival rate of abandoned chicks that were admitted at younger developmental stages (P0 and P1) than those in later stages (P2 to Blue). When the outcome curve of chicks hatched at SANCCOB is evaluated (Fig 5A), it becomes evident that nearly all mortality occurs within the first 30 days of hand-rearing, before the chicks reach approximately 750 g of body mass (see Fig 4). A similar pattern has been observed in wild penguin populations, where chick mortality due to factors other than starvation usually decreases significantly after 30 days of age [17,27,34,35]. It is therefore clear that this is the most vulnerable period of hand-rearing, and future efforts to improve rearing protocols should emphasize the husbandry and medical care during this critical interval.

For chicks removed pre-emptively, i.e. without a history that would suggest a compromised health status, the release rate achieved with SANCCOB’s hand-rearing was remarkably high (93%). This is a release rate equivalent to that of adult African penguins that were admitted due to oiling [36]. For the chicks that hatched at SANCCOB, the overall release rate was 57%; however, this rate increased rapidly over the years following the establishment of the Chick Rearing Unit, and in 2016 release rate was 87%. The progressive increase in the release rate over the years can be attributed to an outbreak of *Cryptosporidium* sp. in 2012 and 2013 (Hurtado et al., in prep.) and to the fine-tuning of rehabilitation protocols and procedures. It is not possible to quantify the effect of each specific change to the rehabilitation protocols had on the release rate because this fine-tuning process was a fluid and dynamic process, where the chick rearing procedures were continually re-evaluated by the staff and the protocols were cumulatively adjusted aiming to improve animal welfare, prevent health problems, and promote a healthy growth and development. As such, this decision-making process was not strictly data-based and involved some degree of subjectivity; however, it was based on the combined evaluation and discussion of the rehabilitators and veterinarians in weekly staff meetings, and was therefore a consensus-building process by experienced professionals. The high release rates achieved in the latest years attests to the efficacy of this fine-tuning approach. With the exception of a small number of chicks admitted due to injury or deformity, the 2016 release rates are superior to the fledging rate of wild African penguins, which under “normal” circumstances will fledge on average 61% of the hatched chicks [37], corroborating that the hand-rearing protocols evaluated in this study can be considered to produce satisfactory results.

Previous studies have shown that post-release survival and breeding success of hand-reared African penguin chicks is comparable to that of their cohorts naturally reared by their parents in the wild [9,10]. Due to concerns on the negative impacts that flipper banding can have on penguins [38], SANCCOB stopped placing metal flipper bands on African penguins before they were released since 2010, which precludes analyses on the post-release outcomes for the dataset presented in this study. Since 2015, however, SANCCOB has systematically implanted microchips in hand-reared penguins prior to release, and this will allow for future analyses of the post-release survival and breeding success and how hand-rearing protocols can be further advanced to improve these parameters.

Although we consider SANCCOB’s efforts to incubate and hand-rear African penguins a success story, it must be considered that this is a drastic and expensive strategy that does not address the primary conservation threats challenging the species in the wild. The African Penguin Chick Bolstering Project was conceived as an emergency measure to slow down the collapse of the species’ population in the wild [7,11], and will only constitute a valuable contribution to its conservation if other longer-term conservation strategies are put into place to address the species’ conservation threats (e.g. fisheries management, marine protected areas, predator management) [25,26,31,39]. This approach might not be a cost-effective strategy for other avian species that are more sensitive to handling, that have a greater tendency to become imprinted or that do not adapt after being released. We would therefore caution against interpreting the success of the African Penguin Chick Bolstering Project as a basis to recommend this approach be broadly employed for the conservation of other seabird species.

Nonetheless, we do consider there may be potential for the implementation of chick bolstering programs for other species with small populations and for which large-scale breeding failures or nest desertion could have considerable long-term demographic impacts. For instance, two species that might meet these criteria are the Galapagos penguin (*Spheniscus mendiculus*) and the yellow-eyed penguin. The strong El Niño events of 1982–1983 and 1997–1998 were followed by crashes of 77% and 65% of the Galapagos penguin population, respectively, with virtually all nests having been abandoned in those years [40,41]. Similarly, the yellow-eyed penguin population in New Zealand’s South Island has faced large-scale breeding failures or mortality events in several years (1938–1939, 1941–1942, 1989–1990, and 2013) [42,43]. Both Galapagos and yellow-eyed penguins are currently classified as Endangered with global populations of less than 2,000 breeding pairs [44,45], and such mortality events and breeding failures have had significant long-term impacts on the conservation of these species. In this context, the removal and hand-rearing of abandoned eggs and chicks could be a strategy to mitigate the demographic impact of these episodes of large-scale adult mortality and/or nest desertion. This strategy is equivalent to the hand-rearing of penguins orphaned during oil spills [9,22], and could be justified on ethical grounds considering our responsibility to mitigate the population decline experienced by these species as a result of human activities. In this context, the knowledge developed through the hand-rearing of African penguins could provide a basis from which to develop the procedures and protocols that can be used for other penguin species (and other seabirds).

## Supporting information

S1 File Recommendations for the incubation and hand-rearing of African penguins (*Spheniscus demersus*). (PDF)

S2 File Compressed file containing the raw data in CSV format (comma-separated values). (ZIP)
