# Supplementary material for: From incubation to release: Hand-rearing as a tool for the conservation of the endangered African penguin
Source: PLoS One. 2018 Nov 7;13(11):e0205126. doi: 10.1371/journal.pone.0205126 (PMC6221267; doi:10.1371/journal.pone.0205126)
Supplement: S1 File — (PDF) [file pone.0205126.s001.pdf]

S1 FILE

Recommendations for the incubation and hand-rearing of African penguins (*Spheniscus demersus*)

The Southern African Foundation for the Conservation of Coastal Birds (SANCCOB) was founded in 1968, and since then it has rehabilitated over 95,000 seabirds. SANCCOB's Chick Rearing Unit was officially opened in November 2011, and until December 2016 it had received nearly 700 eggs and 3,000 chicks. This document details the protocols and recommendations for the incubation of eggs and the hand-rearing of African penguin chicks as employed during this period at SANCCOB.

It should be noted, however, that these protocols and procedures are constantly being re-evaluated and improved, and will certainly change in coming years. Furthermore, it should be born in mind that the rehabilitation protocols are adapted to the logistical limitations and practical realities and to the health status and condition of the birds admitted at SANCCOB. Therefore, these protocols will likely need to be adapted if they are to be implemented by other organisations.

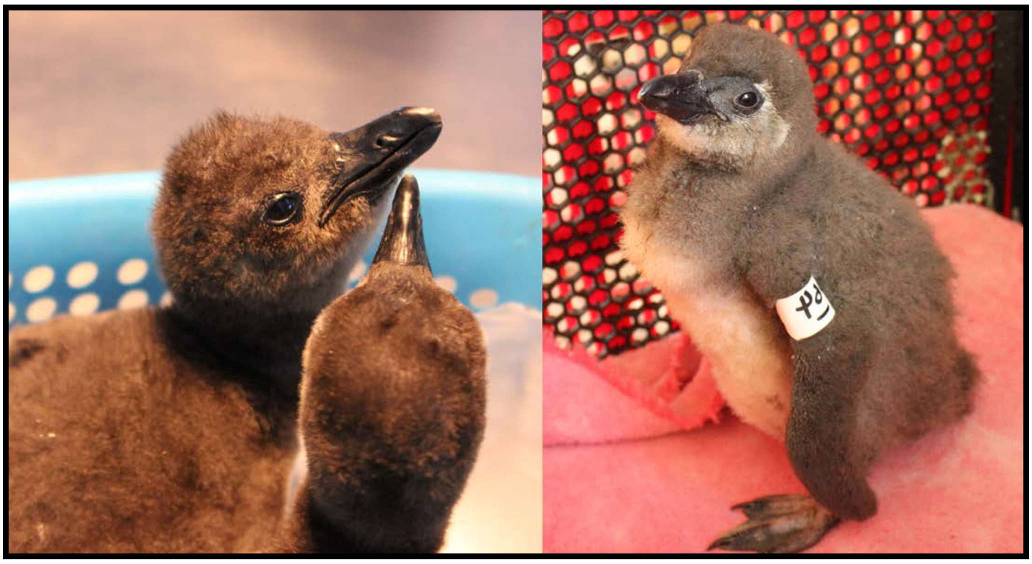

This document is a supplementary material to the following publication:

Klusener R, Hurtado R, Parsons NJ, Vanstreels RET, Stander N, van der Spuy S, Ludynia K. 2018. From incubation to release: Hand-rearing as a tool for the conservation of the endangered African penguin. *PLOS One*.

Table of Contents

1. TRANSPORTATION OF EGGS AND CHICKS 3

2. ADMISSION OF EGGS 3

3. EGG INCUBATION 5

4. ADMISSION OF CHICKS 7

5. HOUSING 8

6. FOOD PREPARATION 13
   6.1. Preparation of Darrow's solution 13
   6.2. Preparation of fish formula 14
   6.3. Preparation of fish fillets and fish tails 15
   6.4. Vitamin supplementation 16

7. FEEDING REGIMEN 16
   7.1. Monitoring weight gain 20
   7.2. Overfeeding 23
   7.3. Emaciation regimen 23
   7.4. Supplemental hydration 24

8. FEEDING PROCEDURE 25

9. PRE-RELEASE CONDITIONING 29

10. RELEASE CRITERIA 30

11. HYGIENE 31
   11.1. Foothbath 31
   11.2. Personal hygiene 32
   11.3. Cleaning syringes and tubes 33
   11.4. General cleaning 33

12. GENERAL VETERINARY CARE 34

13. DETAILS ON THE COMMERCIAL PRODUCTS 35

1. TRANSPORTATION OF EGGS AND CHICKS

Eggs are placed horizontally in foam-cases designed for egg transportation and placed in a portable incubator set at 30 °C. Clean towels are placed on the base of the incubator and around the foam-cases to protect the eggs from crushing/rupturing internal veins (Figure 1a). It is essential to handle eggs very carefully and rotate or move them abruptly, to avoid damage to the embryo and blood vessels. Chicks from different stages (P0 to P4) are accommodated (max. of 3 individuals) in cardboard boxes (58 x 36 x 43 cm) with holes for ventilation and towels on the bottom (Figure 1b).

Eggs and chicks are transported to the Chick Rearing Unit (CRU) on the same day. A pick-up vehicle with canopy is used to transport the birds, aiming to keep them separate from the driver and also to provide them with a sheltered and comfortable environment. Portable incubators and bird transport boxes are tied to avoid accidents, and good ventilation is provided by opening side windows (Figure 1c).

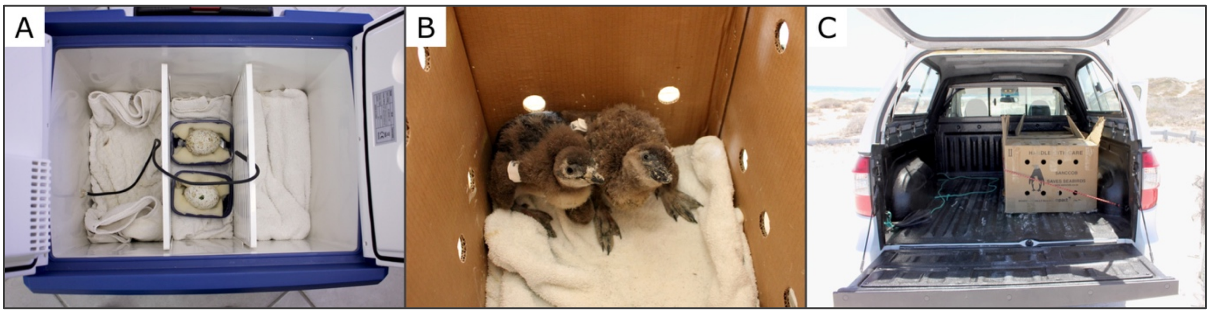

Figure 1. Transportation of eggs and chicks. Legend: (A) Portable incubator used to transport eggs; (B) Chicks transportated in a cardboard box; (C) Adequate bird transportation.

2. ADMISSION OF EGGS

1) Identification: an individual number (marked with pencil on the shell) and an individual sheet are assigned to each egg;
2) Morphometrics: eggs are weighed (precision 0.01 g) and their length and breadth are measured with a calliper (precision 0.1 mm) (Figures 2a and 2b);

3) Candling: to evaluate shell quality, egg shell cracks and flaws, yolk quality and mobility, embryo stage and determine air cell formation and position (Figure 3);
4) Incubation: after the admission process, eggs are placed in a pre-heated incubator (Compact S84 model 8015 – Grumbach®, Mücke, Germany) set at 36.5 °C and 45% humidity.

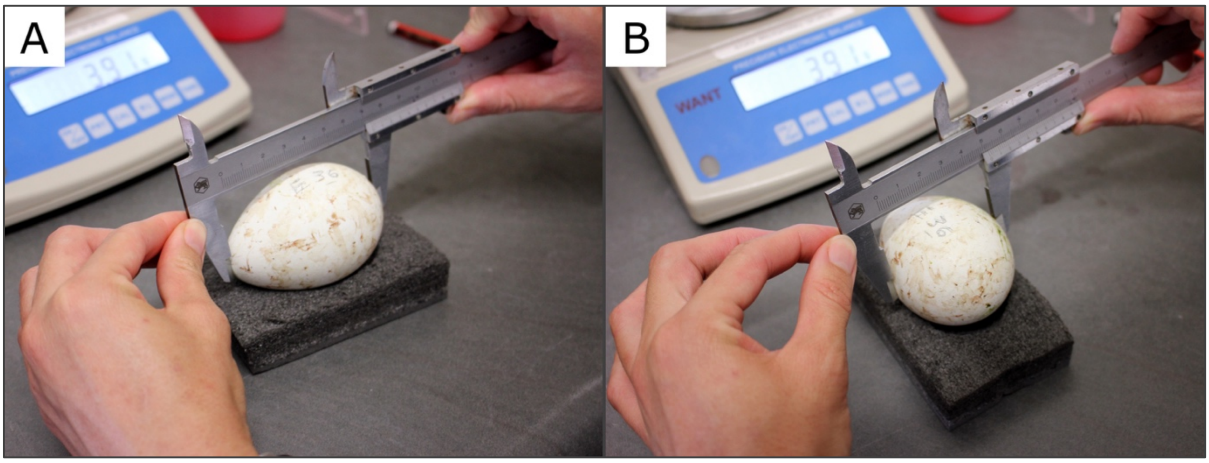

Figure 2. Measurement of eggs. Legend: (A) length and (B) breadth.

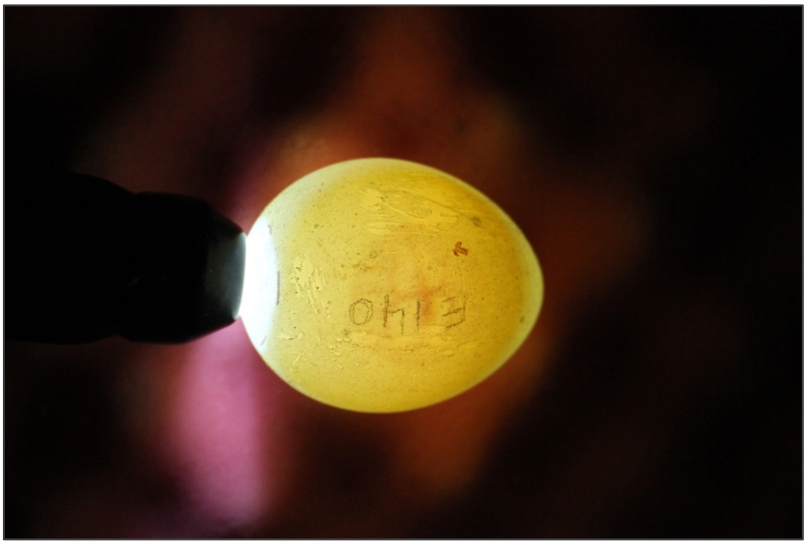

Figure 3. Egg candling to evaluate shell quality, cracks and flaws, yolk quality, embryo stage, and determine air cell formation and position.

3. EGG INCUBATION

Eggs are gently placed horizontally on the rotating tray and not touching other eggs in an incubator (Compact S84 model 8015 – Grumbach®, Mücke, Germany) set at 36.5 °C and 45% humidity (Figure 4a). To ensure embryonic development, the incubator is set to continually rotate eggs widthways (complete rotation every 2 hours), and eggs are manually rotated 180° lengthways every 24 hours, to help appropriate development of vascularization in the egg.

Eggs are weighed daily (precision 0.01 g) and candled (High Intensity Egg Candler – Titan Incubators®, Minety, UK) every 7 days. Candling is the primary method for monitoring embryonic development and movements. During incubation, development of the embryo and its extraembryonic membranes should be monitored to the extent possible, based on visibility. The progression of the air cell is also tracked by tracing its margin on the shell with a blunt pencil during candling. It is worth mentioning that when the eggs are too soiled it makes candling difficult especially on the early stages of incubation; however, washing the eggs is not recommended.

Digital egg monitors (Buddy® - Avian Biotech, Tallahassee, USA) can be helpful to monitor the embryo's heartbeat, but there are limitations to be considered. In our experience, the sensor fails to detect heartbeats until after the Day 12 stage and it is not reliable if the egg shell is too soiled. This type of sensor should therefore not be used as a single method to determine if an egg is not viable and needs to be discarded.

Once the egg externally pipped (usually approximately 38 days after being laid), it is placed in a labelled plastic container with soft paper towels (Figure 4b) and moved to an incubator (Compact S84 model 8015 – Grumbach®, Mücke, Germany) set at 36.5 °C with 60% humidity until hatching. This higher humidity setting is important to prevent egg membranes from drying too fast. At this point, both manual and automatic rotation of the egg are interrupted.

Assisted hatch is performed when a chick did not hatch 48-50 hours after having first externally pipped. The most common hatching difficulty observed for African penguin chicks is the incorrect positioning of the chick inside the egg. When performing an assisted hatch, hands should be thoroughly washed and sterile instruments used to prevent transmission of pathogens to the chick. The chick should be examined and an evaluation of the problem

must be assessed before beginning to assist (see detailed recommendations provided by Tollini 2007).

During incubation, African penguin eggs usually lose about 13 to 15% of weight by water evaporation through the pores of the eggshell. Because this is a physical process, this rate is relatively constant regardless of the stage of embryonic development. Under artificial incubation conditions, this weight loss is managed by controlling the humidity in the incubator: if the humidity is too high, weight loss will be insufficient, and if humidity is too low will result in excessive egg weight loss.

Cracked or punctured eggs are often still hatchable if carefully repaired. If not repaired, they are vulnerable to infection and excessive weight loss. Clear nail polish is quick-drying and has been used successfully for African eggs, but it may contain volatile solvents that are potentially toxic to the embryo. White glue, although slow-drying, is an excellent nontoxic material for repair that also provides some structural support. It is worth noting that higher settings of incubation humidity may be also be used to correct excessive weight loss in eggs with cracked or punctured shell.

Eggs were discarded if there was any oozing of the egg or bad smell present, if their yolk showed no development after 14 days of incubation, if an egg did not hatch after 2 weeks past the estimated hatching date (especially if the egg has been incubated since the yolk or small embryo stage), or if after 7-13 days the eggs presenting one of the following characteristics: a dark black spot was seen instead of embryo without surrounding veins, there was a solid dark red ring around a dead embryo with no vein network, an air bubble was seen floating in the dark part of the egg (often near to the air sac), or the dark part of the egg appeared liquid and not a solid embryo. Whenever feasible, discarded eggs were examined to determine if an embryo was present or not, and to establish the embryonic stage of development: early (very small embryo, approximately 2 cm, large head with large eyes, poorly developed limbs), mid (head relatively well-proportioned to the body, limbs well developed, no fluff), late (fully developed, fluff present, about to hatch), or deceased during hatching.

When the chick hatches (and is completely detached from its egg), it is checked for abnormalities, weighed (precision 0.01 g), placed in a container with clean soft paper towels, and then relocated to a pre-heated incubator (Compact S84 model 8015 – Grumbach®, Mücke, Germany) set at 36.5 °C and 45% humidity for 12 hours to fluff out

(Figure 4c). Starting five hours after hatching, 1 mL of probiotics (a concentration of 1 g Kyron Protexin® in 100 mL water) is administered (using a rubber feeding tube attached to a syringe) every 3 hours for the first 24 hours (except overnight, after the 21:00 and before the 06:00 am feeds).

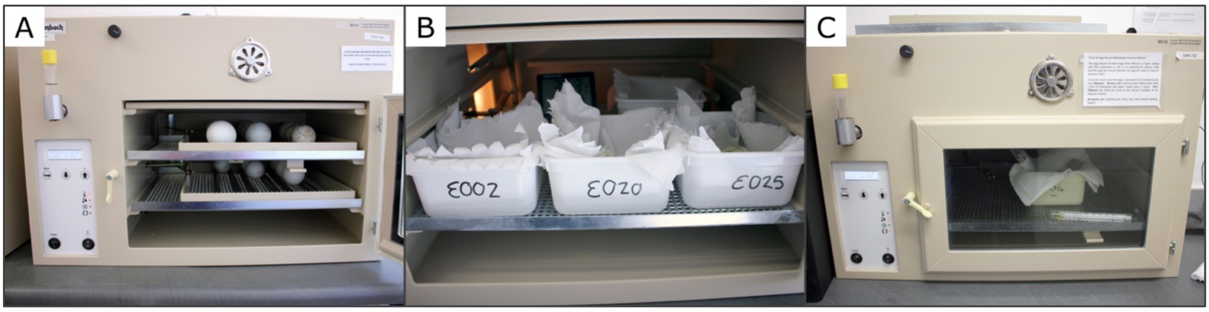

Figure 4. Stages of egg incubation. Legend: (A) egg incubator; (B) hatcher incubator; (C) dry-out incubator.

4. ADMISSION OF CHICKS

1) Age determination: chicks are classified into stages of development (P0, P1, P2, P3, P4 or Blue) based on Barham et al. (2008) and Sherley et al. (2014);
2) Identification: an individual number (leg tag or flipper band) and an individual card are assigned to each chick. A numbered tape (Transpore™ – 3M, St. Paul, USA) wrapped around each leg for P0, P1 and small P2 (Figure 5a), or a numbered paediatric wristband for over larger P2 put on each flipper (Figure 5b). Tags and bands must be checked daily for tightness, because the chicks will grow quickly;
3) Weighing: scale precision 0.01 g or 0.02 kg, depending on chick size;
4) Physical examination: standard veterinary procedure comprising the evaluation of hydration, mucosae, signs of pain, external lesions, locomotor apparatus, pulmonary/air sacs auscultation, etc.;
5) Feather dusting: sprinkling of the chick's back and abdomen with insecticide powder (Karbadust® – Efekto, Benmore, South Africa) on over P2 stage. For P0 and P1 with ectoparasites, fipronil (Frontline®, Merial Inc., Duluth, USA) is sprayed onto a swab and then dabbed it down the back of the chick;
6) Birds that are alert (i.e. holding their head up) receive oral hydration based in Darrow's solution according to their mass and developmental stage:

a. P0 and P1 chicks receive up to 5 mL of a mixture of Darrow's solution and fish formula (50:50);
b. P2 and P3 chicks weighing less than 500 g receive 10-20 mL of Darrow's solution;
c. Chicks weighing 500 g or more receive up to 60 mL of Darrow's solution and an initial dose of deworming drugs (ivermectin 0.2 mg/kg and praziquantel 7.5 mg/kg, same dose to be repeated after 14 days).

7) Record keeping: the individual card is populated with all the relevant information about the bird, such as location of rescue, date, reason for admission, age, initial treatment, overall condition, feeding regime, blood results, etc.;
8) Triage: chicks are allocated to a stage of the rehabilitation process that is consistent with their age group, size and health status.

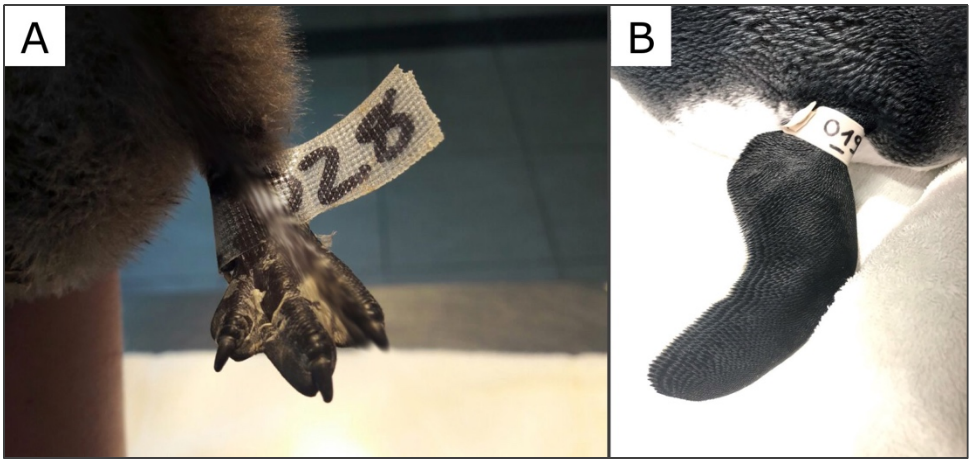

Figure 5. Marking of chicks. Legend: (A) numbered tape wrapped around leg for P0, P1 and small P2 chicks; (B) paediatric wristband tag placed around flipper for larger chicks.

5. HOUSING

Once hatched, P0 chicks are housed individually in plastic pots (14 cm diameter, 13 cm height) with clean soft towels and transferred to a human incubator (Caleo® – Dräger, Telford, USA) set at 33 °C and 45% humidity for 5 days (Figure 6a). P0 and P1 chicks received from the wild are also housed in the same manner. Depending on the size of the pot and the size/behaviour of the chick, a small plush toy may be added to provide company and shelter, and also to stimulate appetite (due to the natural food competition between

siblings) (Figure 6b). From days 6 to 8 after hatching (72 hours in total), the incubator temperature is lowered to 30 °C. It should be noted that these temperature settings are based on SANCCOB's long-term experience, however there is some individual variation. Some chicks will show signs of discomfort (open mouth breathing, trembling, etc.) and may need a higher or lower temperature; therefore, it is important to regularly monitor the chicks' behaviour and posture to adjust temperatures accordingly.

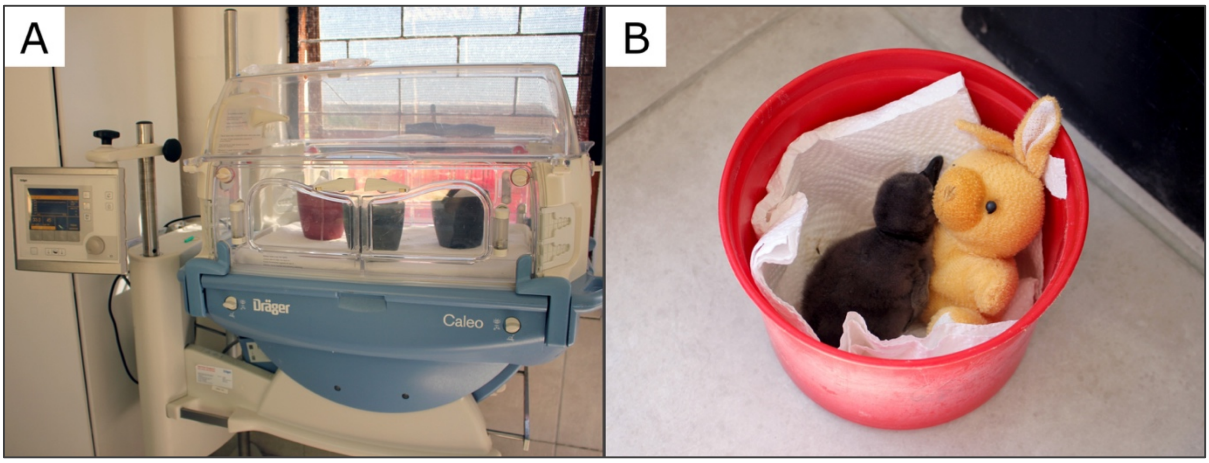

Figure 6. Housing of hatchlings. Legend: (A) hatchlings in human incubator; (B) chick housed in a plastic pot with a small plush toy.

After day 8 (or when body mass surpasses 100 g), the chick (now P1 stage) can be transferred to a brooder crate. The brooder crate (plastic material, 40 x 33 x 30 cm) is set up with towels on each side of it and also on the base, acting as a soft and absorbent substrate. A plush toy is also provided. The brooder crate is kept under an infrared heat lamp, and a folded towel is used to block excess heat for half of the crate (Figure 7). While in the brooder crates, chicks may be kept individually or in pairs if both are healthy and from the same cohort (same nest or area, removed on the same day). It should be noted that overcrowding can lead to overheating chicks and also increase the likelihood of transferring disease. The brooder crate temperature should always be regularly checked, and chicks should be closely monitored for signs of heat or cold stress.

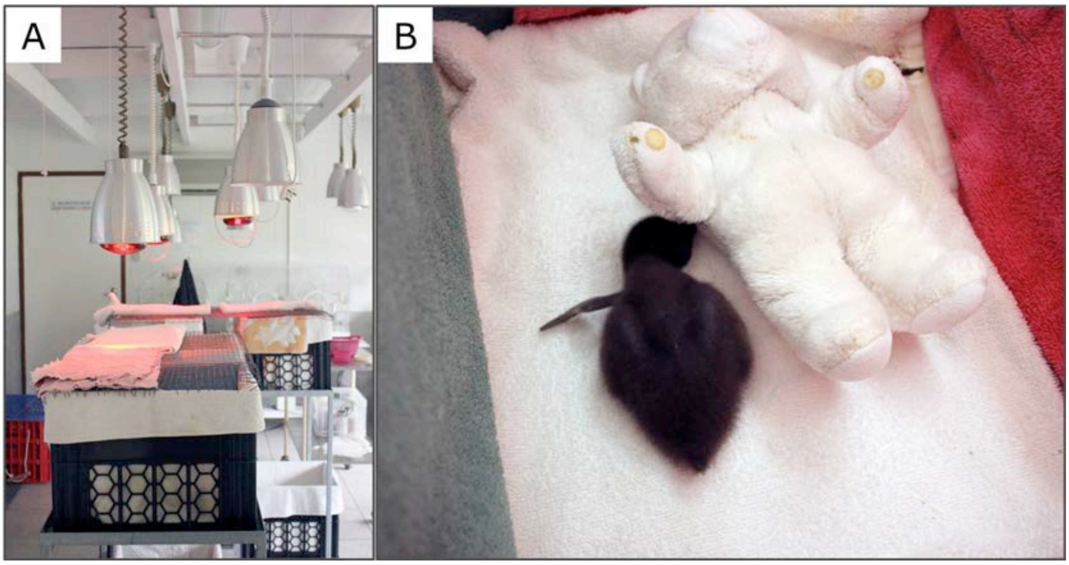

Figure 7. Brooder crates. Legend: (A) brooder crates under heat lamps; (B) chick sleeping with a plush toy.

When the chick reaches 500 g, it can be moved to the large crate (plastic material, 70 x 50 x 50 cm, towels on the bottom) with one other chick (of similar age) in order to allow socialization (if the clinical condition allows it) (Figure 8). If the chick is kept alone in a large crate, a plush toy is always provided. A towel is used to partly cover the crate, which is kept in a well-ventilated room (18 to 21 °C); during days of good weather the crates can be taken outside.

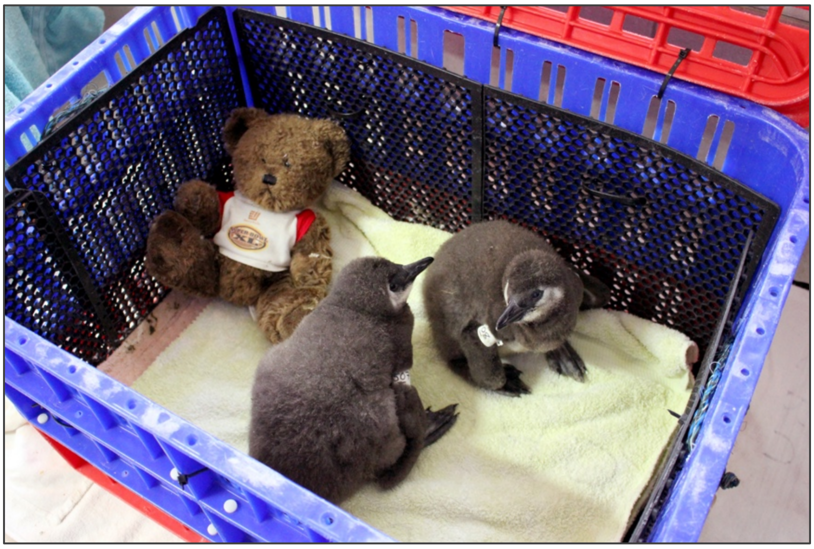
Figure 8. Chicks of the similar age and size sharing a larger crate.

When the chick reaches 1.5 kg, it is transferred to the Nursery Unit, where it will be housed in an external pen during the day (Figure 9a) and an internal pen during the night (or during rainy or excessively cold days) (Figure 9c). Both the external and internal pens have rubber matting to prevent abrasion injuries, and birds are divided into different pens according to their health status (e.g. “healthy”, “intermediate”, “sick”), if necessary. The external pen has artificial nests (“kennels”) to provide shelter/hideout (Figure 9b), whereas heat lamps and plush toys are provided in the internal pens (Figure 9d).

When the downy plumage had been mostly lost (some P4 and all Blue stages), they are transferred to the general rehabilitation facility (details on Pre-release conditioning). At this stage, they were permanently housed outdoors with pool access, in large groups (occasionally sharing the pen with juvenile and adult African penguins) (Figure 10).

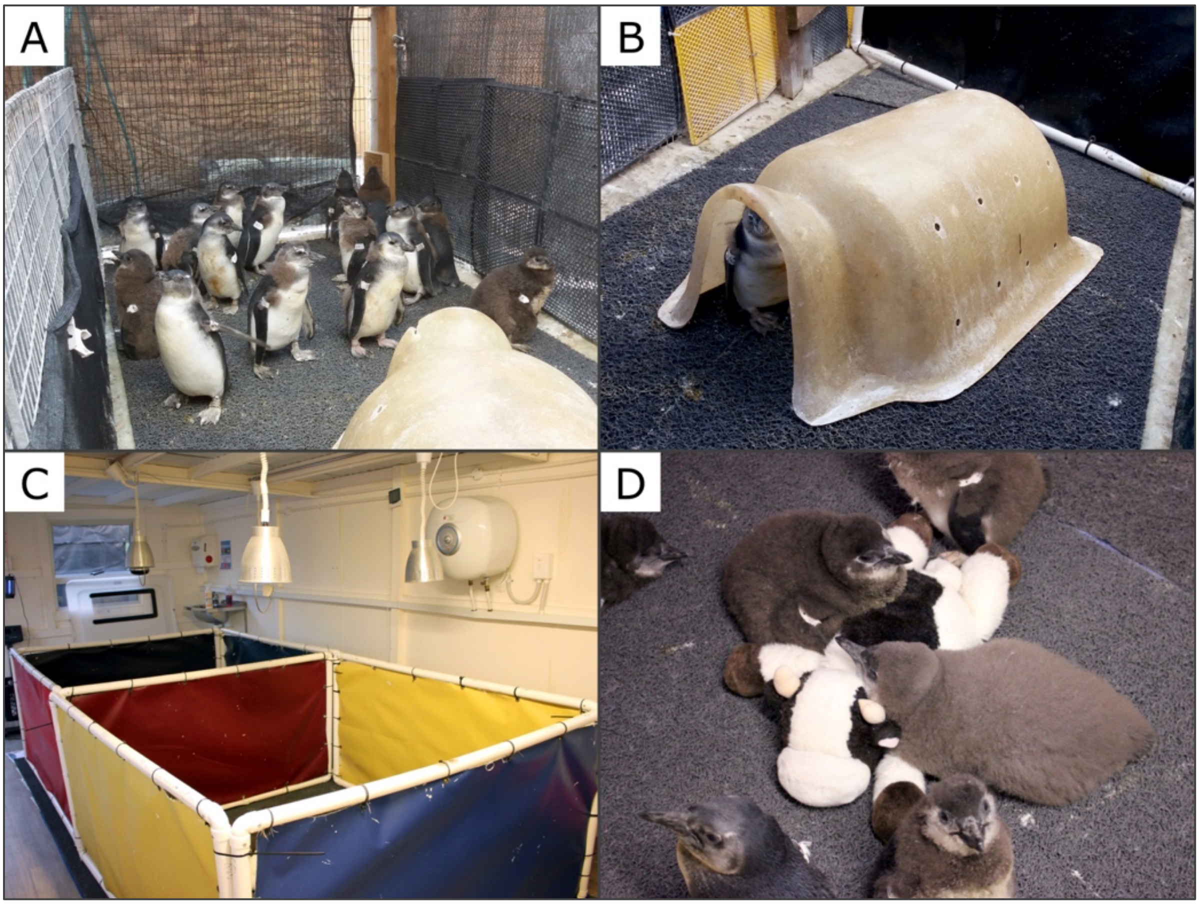

Figure 9. Housing at the Nursery Unit. Legend: (A,B) external pens; (C,D) internal pens.

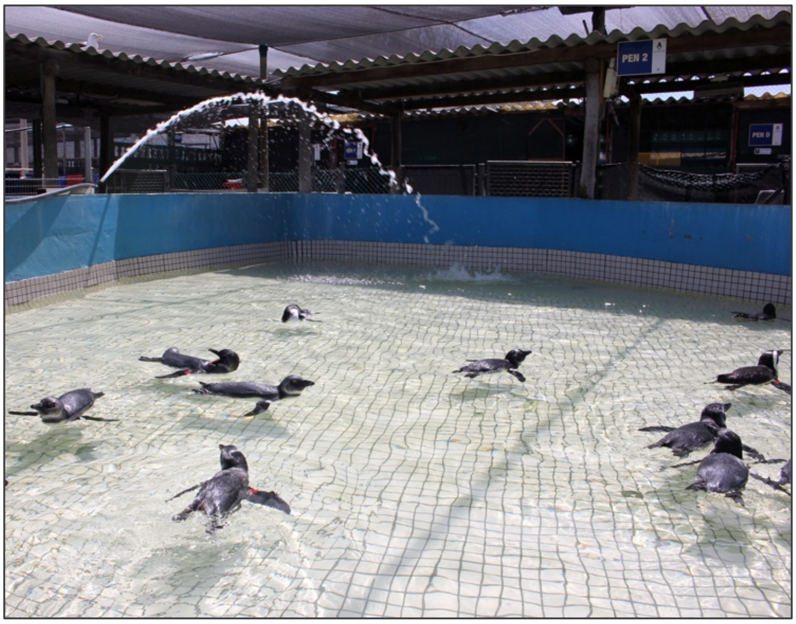

Figure 10. Pool pen at general rehabilitation facility.

It should be noted that providing adequate footing is essential to prevent deformities, injuries and pododermatitis (“bumblefoot”). Clean, dry towels are the most commonly used substrate in the brooder and large crates. Roughly textured or frayed towels should not be used because of the risk of injuries resulting from snagged toenails, neck entanglement or attempts to climb. It is equally important that the substrate is not too smooth to prevent the chick's feet from sliding out underneath it. Rubber matting can also be used in addition to towels to provide a firm surface.

Plush toys are useful to provide comfort to penguin chicks, since these are highly social birds that tend to seek company of other chicks. Selection of the plush toys should be mindful with regards to detaching areas, quality of the material, comfort and size. Penguin chicks should not be kept alone without a plush toy, even when they are old enough to be in a large crate.

A room temperature of 18 to 21 °C is generally recommended for penguin chicks weighing more than 500 g. However, the behaviour of the chicks should be checked as often as possible to adjust the temperature if necessary. Symptoms of hyperthermia (overheating): lethargy, loss of appetite, panting, and extension of feet and flippers, and slight dehydration. Overheating can be problematic for chicks at any age but may quickly become life-threatening in very young chicks unless corrective measures are taken. Symptoms of hypothermia (underheating): shivering, huddled against the side of the crate,

feet and flippers are tucked in and cold to the touch, and slow response to a feeding stimulus. When adjusting a heat lamp, always make sure the lamp and the bulb are secure and will not fall or change position. Heat lamps should be adjusted to the chicks' individual needs, and should be placed in a manner that part of the crate is shielded from excessive heat (for example, using towels to cover part of the crate). Appropriate ventilation is also essential, as a constant flow of clean and dry air can be critical to provide a comfortable environment and prevent respiratory diseases. Crates or pens should never be excessively closed or blocked, and fans can be used to force air circulation.

Because penguins are highly susceptible to avian malaria, controlling mosquitoes in the facilities is essential. Netting all facilities is the most effective measure, but additional strategies should also be employed. Roll-on insecticides (diethyl-meta-toluamide 35% roll-on stick) can be applied locally to the birds' heads to help prevent flies and mosquitoes as long as they do not interfere with plumage waterproofing. Adhesive fly traps and other insect control measures (e.g. insect repellent electric diffusers) are also routinely employed at SANCCOB.

6. FOOD PREPARATION

The fish used during the whole process of the chick-rearing is the Southern African pilchard (*Sardinops sagax*). The quality of all products purchased for feeding to penguins should meet human food standards. Fish must be properly frozen and stored until used (-18 to -30 °C, up to 6 months), and cannot be defrosted for more than 24 hours; once defrosted, fish should not be refrozen. Details on the composition of commercial products mentioned in this section are provided at the end of this document.

6.1. Preparation of Darrow's solution

Darrow's solution is prepared by diluting the 50 mL of the commercial solution in 1000 mL of tap water, and then adding 12 g of Protexin®.

6.2. Preparation of fish formula

Fish formula is prepared by blending the following ingredients for 5-7 minutes until a drinking yogurt consistency is achieved, then straining the mixture twice on a sieve (1 mm mesh):

- 880 g fish (after removing tail and fins)
- 600 mL tap water
- 5 capsules Edelweiss® Large Fish Eating Bird Formula
- 600 mg thiamine
- 3 g Brewer's yeast
- 2.5 g Calcium gluconate
- 5 mL AquaMarine® oil
- 5 g Protexin® Soluble
- 5 g Cani-cal®
- 3 capsules vitamin E (400 I.U. per capsule)

The fish formula can be prepared pure (as per recipe above) or it can be diluted in Darrow's solution to produce a more liquid consistency for younger or weaker chicks. The most commonly-used dilutions are 25:75 (25 mL of formula are mixed with 75 mL of Darrow's solution), 50:50 (50 mL of formula are mixed with 50 mL of Darrow's solution) and 75:25 (75 mL of formula are mixed with 25 mL of Darrow's solution).

After having been prepared, the fish formula must be refrigerated (2 to 8 °C) and used within 12 hours for chicks under 1.5 kg (Chick Rearing Unit) and within 24 hours for chicks over 1.5 kg (Nursery Unit); after 24 hours, the formula must be discarded. Because the fish formula is stored in the refrigerator, it needs to be warmed prior to being administered to the birds. The fish formula should not be heated directly on a stove or microwave, but instead it should be drawn up in syringes which are then placed in warm water to slowly heat up (Figure 11). If it is not given to chick after having been heated, the fish formula should be discarded (do not return to the refrigerator and re-use).

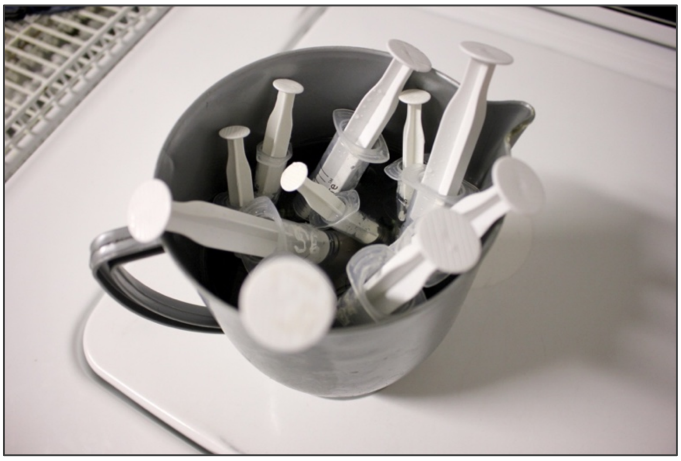

Figure 11. Syringes placed in warm water to slowly heat up.

6.3. Preparation of fish fillets and fish tails

Fish fillets are offered to chicks weighing 500 g or less, and fish tails are offered to chicks weighing from 500 g to 1000 g. Whole fish is offered to chicks weighing over 1 kg. Fish fillets are prepared by slicing away the spine by cutting lengthwise along one side of the fish parallel to the backbone; any scales, viscera and bones should be removed (Figure 12a). Fish tails are prepared by cutting the fish diagonally from behind its anal fin to the dorsal fin, then removing the anal fin, viscera and the head; depending on the size of the chick, it is possible to prepare smaller (Figure 12b, top) or larger fish tails (Figure 12b, bottom).

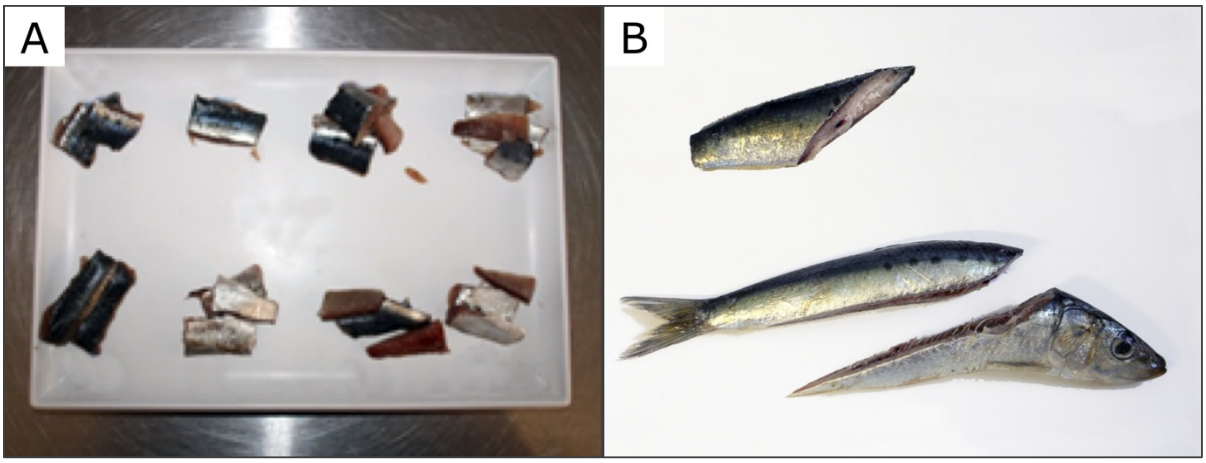

Figure 12. Preparation of fish. Legend: (A) fish fillets; (B) small and large fish tails.

6.4. Vitamin supplementation

Daily vitamin supplementation is provided for chicks with a body mass equal to or greater than 1.1 kg. Vitamin tablets are inserted on fish's mouth or gills, and the fish is then fed normally to the birds.

- Monday: 1 tablet of Edelweiss® Large Fish Eating Bird Formula
- Tuesday: 1 tablet of Portfolio® Multivitamin orange
- Wednesday: 1 tablet of Portfolio® Vitamin B complex and 1 tablet of thiamine (100 mg).
- Thursday: 1 tablet of Edelweiss® Large Fish Eating Bird Formula
- Friday: 1 tablet of Portfolio® Multivitamin orange
- Saturday: 1 tablet of Edelweiss® Large Fish Eating Bird Formula
- Sunday: 1 tablet of Portfolio® Vitamin B complex and 1 tablet of thiamine (100 mg).

7. FEEDING REGIMEN

During the hand-rearing process, each chick is weighed every day (scale precision 0.01 g) before the first meal of the day to monitor its development and calculate the diet for the day. The feeding regimen is summarized in Table 1.

On the day it hatches, the chick receives only probiotics (see on section "3. Egg incubation"). On the following three days (days 1 to 3), the chick is given fish formula six times a day. A fixed volume of formula is provided on each feed (1 mL in the first day, 3 mL in the second day, 5 mL in the third day), but it should be kept in mind that this is a maximum volume that depends on the behaviour of the chick (small chicks may only accept smaller volumes). On the first day a 50:50 diluted fish formula is administered, whereas on the second day the dilution is decreased to 75:25 and on the third day the fish formula is no longer diluted.

Starting on the fourth day after hatching (or for chicks admitted with less than 100 g of body mass) and until a body mass of 100 g is achieved, the chicks will receive undiluted fish formula six times a day, with a volume equivalent to 10% of the chick's body weight in

each meal. For example, a chick weighing 93 g in the morning will receive six meals, each consisting of 9.3 mL fish formula.

When the chick reaches 100 g of body mass, fish fillets will be introduced in its diet. The number of meals with fish fillets will gradually increase over the first four days (1 meal with fish fillets on the first day, 2 meals on the second day, 3 meals on the third day, and 5 meals on the fourth day), and the quantity of fish will also increase (3 g of fish fillets per meal on the first day, 5 g of fish fillets per meal on the subsequent days). To ensure that an appropriate quantity of food is provided in each meal, the volume of formula will have to be adjusted by deducting the quantity of fish that was provided in the same meal. For example, a chick weighing 114 g on its second day of introduction of fish will receive four meals of formula only (11.4 mL in each meal) and two meals of fish and formula (5 g of fish fillet and 6.4 mL of formula in each meal).

When the chick has completed the transition to the fish and formula diet and is receiving fish fillets on five meals per day, the mass of fish provided in the last fish meal of the day (18:00) does not have to be deducted from the volume of formula. For example, a chick weighing 137 g will receive five meals of fish and formula during the day (5 g of fish fillet and 8.7 mL of formula in each meal), and then at 21:00h it will receive a formula only meal (13.7 mL).

The fish fillet and formula regimen progresses gradually with increasing quantities of fish depending on the chick's body mass (from 5 g of fish per meal for chicks under 150 g, increasing up to 15 g of fish per meal for chicks up to 400 g). When the chick reaches a body mass of 400 g, the quantity of fish is calculated as a percentage of its body mass. For example, a chick weighing 480 g will receive five meals of fish and formula (24 g of fish and 24 mL of formula in each meal), and then at 21:00h it will receive a formula only meal (48 mL).

After a chick achieves a body mass of 500 g, it starts receiving fish tails instead of fish fillets. In each meal, it will receive a fixed volume of formula (30 mL) and a quantity of fish that is sufficient to complete the meal to the equivalent to 10% of the chick's body weight. For example, a chick weighing 860 g will receive five meals consisting of fish and formula (30 mL of formula and 56 g of fish tails).

When the chick reaches 1 kg of body mass, whole fishes and Darrow's solution are introduced to its diet and each meal is no longer calculated based on the chick's body mass.

The volume of Darrow's solution to be administered is the same as the volume of formula. For example, a chick weighing 1 to 1.5 kg will receive: 60 mL of formula at 08:00h, 2-3 whole fishes at 10:00h, 60 mL of Darrow's solution at 12:00h, 2-3 whole fishes at 14:00h, 60 mL of formula at 16:00h, and 2-3 whole fishes at 18:00h.

From this stage onwards, there are minor changes to the feeding schedule and quantities of fish offered in each meal, as well as the administration of water at 14:00h for chicks until they reach the Blue stage (when the pre-release conditioning will be initiated and they will have the opportunity to ingest freshwater while swimming in the pools).

Table 1. Feeding regimen for African penguin chicks.

Period / body mass	Formula / Fluids (per meal)	Fish (per meal)	Time of the day									
			06:00	08:00	09:00	10:00	12:00	14:00	15:00	16:00	18:00	21:00
**Day 1 after hatching**	1 mL max (diluted 50:50)	No	F		F		F		F		F	F
**Day 2 after hatching**	3 mL max (diluted 75:25)	No	F		F		F		F		F	F
**Day 3 after hatching**	5 mL max	No	F		F		F		F		F	F
**Up to 100 g**	10% BW	No	F		F		F		F		F	F
**Day 1 on fish**	10% BW (deduct fish)	3 g fish fillet	F+F		F		F		F		F	F
**Day 2 on fish**	10% BW (deduct fish)	5 g fish fillet	F+F		F		F		F+F		F	F
**Day 3 on fish**	10% BW (deduct fish)	5 g fish fillet	F+F		F		F+F		F		F+F	F
**Up to 150 g**	10% BW (deduct fish)	5 g fish fillet	F+F		F+F		F+F		F+F		F+F	F
**150 – 200 g**	10% BW (deduct fish)	7 g fish fillet	F+F		F+F		F+F		F+F		F+F	F
**200 – 300 g**	10% BW (deduct fish)	10 g fish fillet	F+F		F+F		F+F		F+F		F+F	F
**300 – 400 g**	10% BW (deduct fish)	15 g fish fillet	F+F		F+F		F+F		F+F		F+F	F
**400 – 500 g**	10% BW (deduct fish)	5% BW fish fillet	F+F		F+F		F+F		F+F		F+F	F
**500 – 600 g**	30 mL	10% BW fish tail (deduct formula)	F+F			F+F		F+F			F+F	F+F
**600 – 1000 g**	30 mL	10% BW fish tail (deduct formula)	F+F			F+F		F+F			F+F	
**1 – 1.5 kg**	60 mL	2-3 whole fishes		F		Fish	D	Fish		F	Fish	
**1.5 kg – P4**	60 mL	3-4 whole fishes		D		Fish	F	W		Fish		
**Blue**	60 mL	4 whole fishes		D		Fish				F+F		

Legend: F Formula only; F+F Fish and formula; D Darrow's solution; W Tap water; Fish Whole fish

BW = body weight

7.1. Monitoring weight gain

Weighing each penguin chick on a daily basis is essential to ensure a healthy weight gain. Figure 13 shows the weight curves of healthy African penguin chicks, and Figure 14 represents the daily relative mass change. Table 2 provides the equations used to calculate these curves.

When evaluating the body mass of a penguin chick, it is essential to calculate the percentage of body weight gained or loss (%BW) on a daily basis. Relying on the evaluation of the mass change in grams can be very misleading, and it is easy to misinterpret a change as being less significant than it actually is. It is important to consider, furthermore, that chicks can vary considerably in body size and that it is more important to evaluate its behaviour and body condition rather than expect it to fit on a pre-established weight curve.

Table 2. Equations for the expected body mass and daily relative mass change of African penguin chicks.

Curve	Body mass	Daily relative mass change
Lower bound	$y = \frac{2100}{(1 + 39.38 \times 2.71828^{-0.082x})}$	$3 < x \leq 12 \rightarrow y = 0.027x + 0.028$<br>$x > 12 \rightarrow y = -0.036\ln(x) + 0.15$
Average	$y = \frac{2610}{(1 + 35.21 \times 2.71828^{-0.088x})}$	$3 < x \leq 12 \rightarrow y = 0.027x + 0.0902$<br>$x > 12 \rightarrow y = -0.065\ln(x) + 0.2855$
Higher bound	$y = \frac{3100}{(1 + 31.54 \times 2.71828^{-0.094x})}$	$3 < x \leq 12 \rightarrow y = 0.027x + 0.14644$<br>$x > 12 \rightarrow y = -0.089\ln(x) + 0.40$

Figure 13. General expected body mass curve for African penguin chicks.

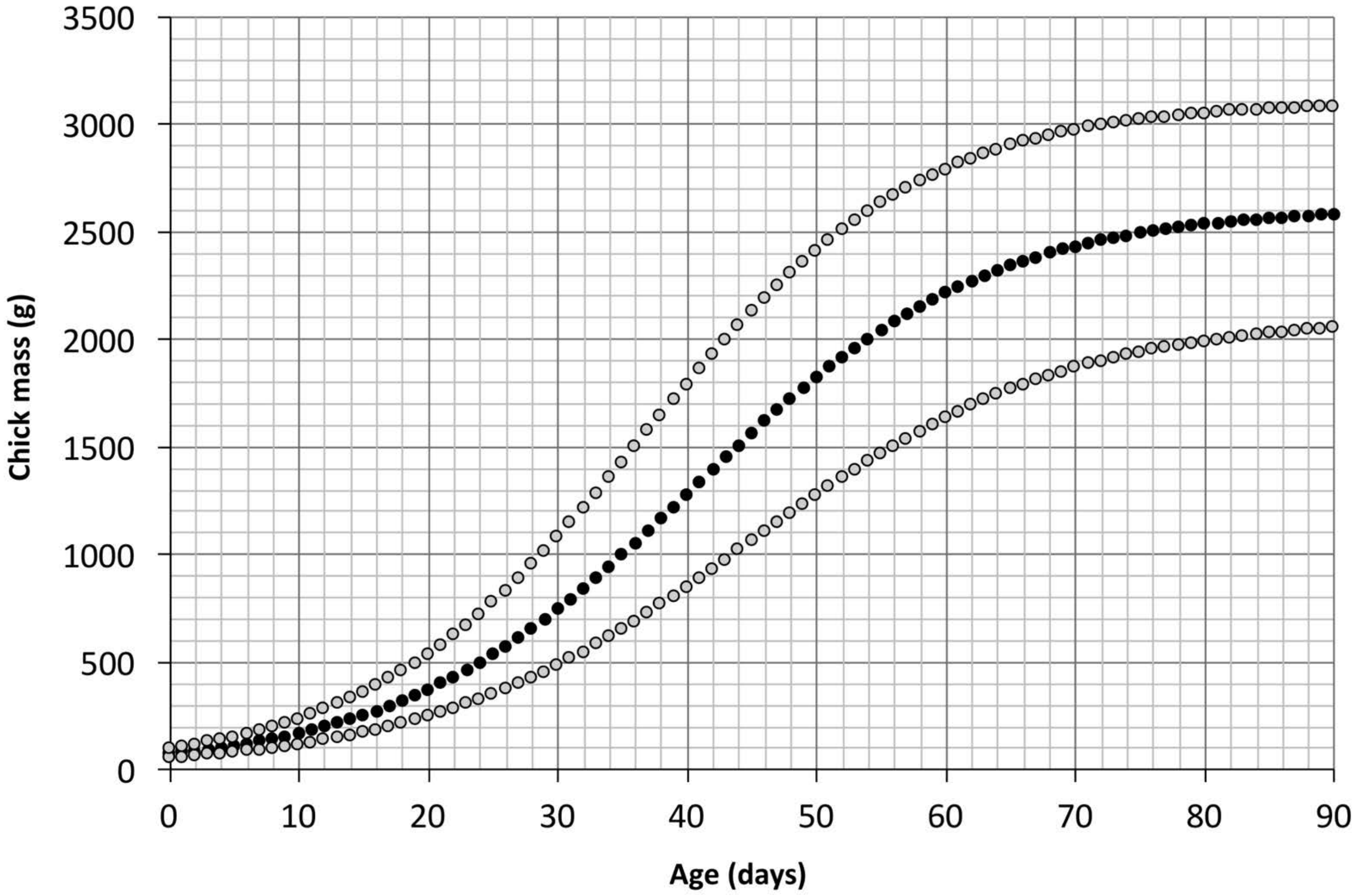

Age	Low	Average	High
**0**	52.0	72.1	95.3
**5**	77.4	110.2	149.7
**10**	114.5	167.3	232.7
**15**	167.9	250.8	356.3
**20**	243.1	369.8	533.3
**25**	346.0	532.5	773.5
**30**	481.2	743.0	1076.4
**35**	649.6	996.9	1425.3
**40**	846.2	1278.0	1787.4
**45**	1058.8	1561.7	2124.8
**50**	1270.7	1822.3	2409.0
**55**	1465.3	2041.6	2628.7
**60**	1631.1	2213.2	2787.6
**65**	1763.6	2339.8	2897.1
**70**	1864.0	2429.3	2970.0
**75**	1937.2	2490.7	3017.4
**80**	1989.1	2531.9	3047.9
**85**	2025.1	2559.2	3067.2
**90**	2049.7	2577.0	3079.4

Figure 14. General expected daily relative mass change of African penguin chicks.

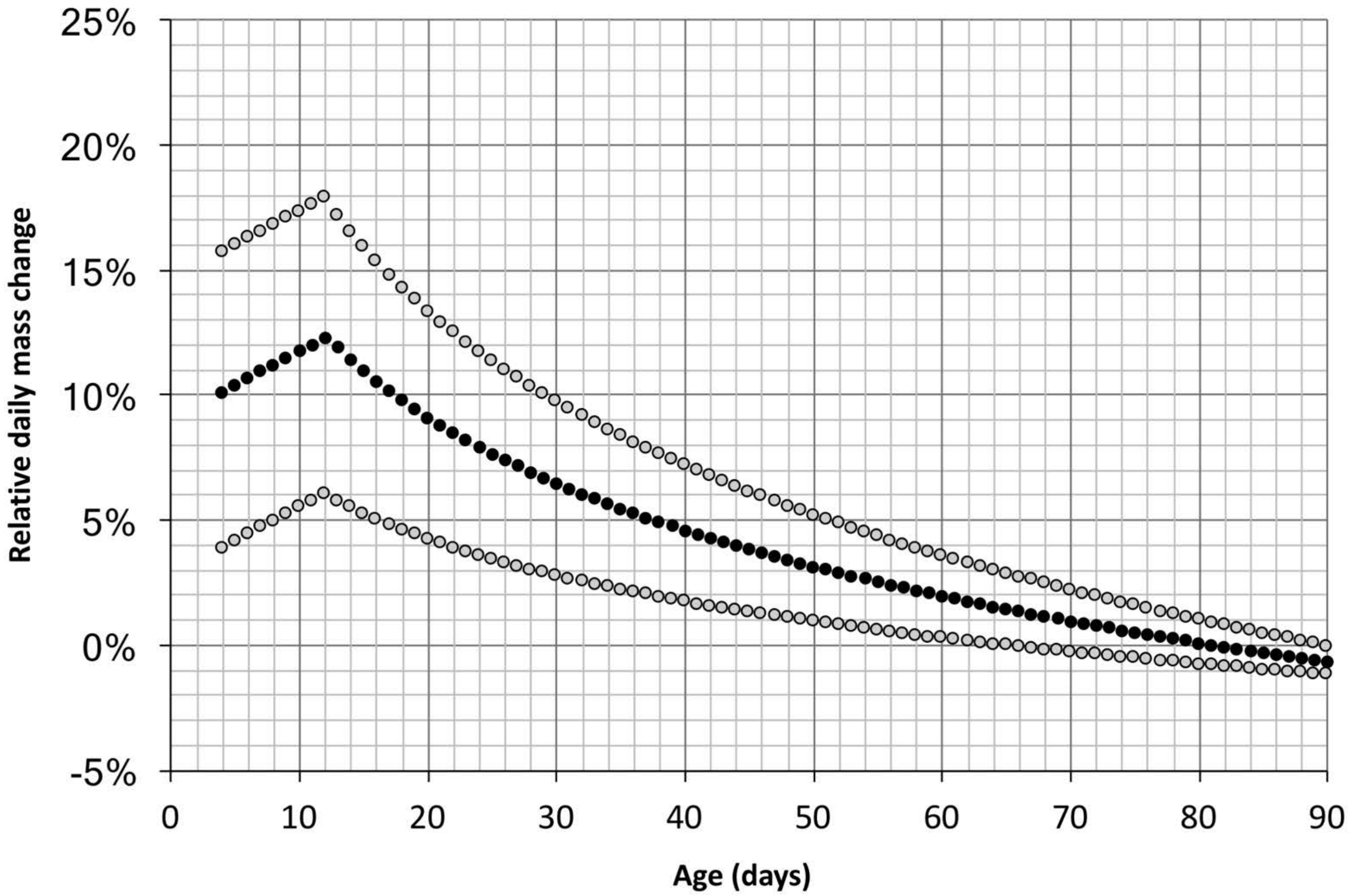

Age	Average
**5**	10.3
**10**	13.0
**15**	12.0
**20**	8.9
**25**	7.2
**30**	6.8
**35**	5.6
**40**	3.9
**45**	3.7
**50**	2.6
**55**	2.6
**60**	0.8
**65**	1.2
**70**	1.8
**75**	0.9
**80**	0.0
**85**	-0.5
**90**	-0.9

7.2. Overfeeding

Even when strictly following feeding protocols and addressing each chick's needs individually, problems from overfeeding may arise. This may be problematic because overfed chicks may lose appetite (and become dehydrated as a result) or, in severe cases, develop deformities (splayed legs). Behaviours associated with overfeeding include lethargy, regurgitation, and loss of interest in food.

It is generally expected that a chick will gain approximately 10% of body mass per day during the early stages of growth, but some fluctuation around this percentage is to be expected. A relatively rapid weight gain is expected during the first three weeks. However, if a chick is gaining more than 13% of its body weight for two days in a row, it is important to adjust its diet to slow the weight gain (unless it is recovering from a period of insufficient weight gain). In most cases, only minor changes are sufficient to reduce this excessive weight gain (for example, removing fish fillets from one or two meals). In more extreme cases, however, it may be necessary to reduce the quantity of food more substantially (for example, reducing meal sizes to 7-8% of body weight instead of the usual 10%). The measures necessary to adjust the weight gain will need to be evaluated on a case-by-case basis considering the chicks' individual history and behaviour.

7.3. Emaciation regimen

Penguins at P4 or Blue stages that were admitted in a condition of emaciation (when the body mass is equal to or lower than 75% of the expected body mass of a healthy individual of the same size; Figure 15) receive a special regimen in order to accelerate their recovery. For this purpose, a special fish formula is prepared by adding an extra 4 g of Protexin® and 4 g of DigestEaze®, and a special feeding and hydration schedule is implemented as detailed in Table 3. Furthermore, these emaciated chicks are kept in crates at a warm/quiet environment and do not have access to the pool (at least for their first and second day after admitted).

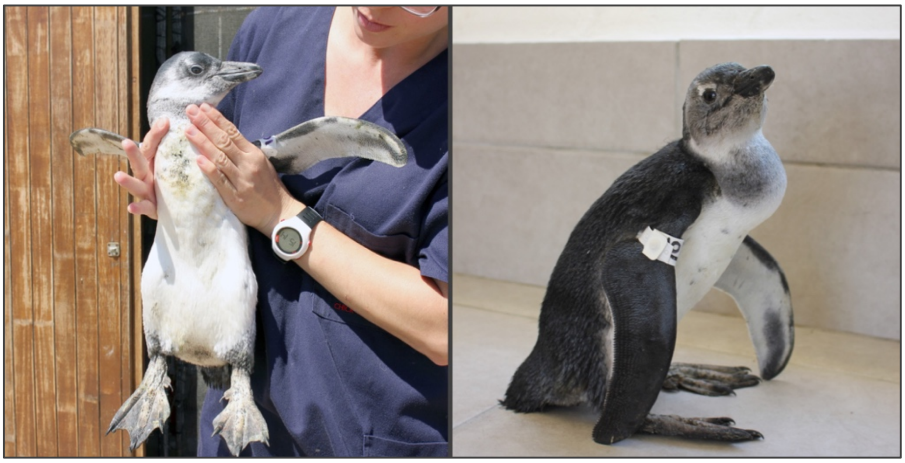

Figure 15. Emaciated Blue stage penguins.

Table 3. Special feeding and fluid regimen for emaciated chicks (P4 and Blue).

Day	08:00	10:00	12:00	14:00	16:00
1	60 mL formula (diluted 25:75)	60 mL Darrow's solution	60 mL formula (diluted 25:75)	60 mL Darrow's solution	60 mL formula (diluted 25:75)
2	60 mL formula (diluted 50:50)	60 mL Darrow's solution	60 mL formula (not diluted)	60 mL Darrow's solution	60 mL formula (diluted 50:50)
3	60 mL formula (not diluted)	60 mL Darrow's solution	60 mL formula (not diluted)	60 mL Darrow's solution	60 mL formula (not diluted)
4	Same as previous day, but start offering fish tails (maximum 2) if bird appears to be normally hydrated				
5	Start on the normal feeding regimen (see "Blue" at the feeding regimen table) if clinical condition has improved and bird appears to be normally hydrated				

7.4. Supplemental hydration

Hydration should be carefully monitored throughout the development of all chicks, and especially when whole fish is introduced into the diet. Symptoms of dehydration include dry appearance of the eyes, shrivelled appearance of the skin on the feet, or thick pasty faeces. The skin along the back will remain "tented up" after pinching it if the chick is inadequately hydrated.

Feeding fish tails or whole fish to a penguin that is dehydrated will only worsen its clinical condition, since the fish will draw substantial quantities of water into the digestive tract, slowing or halting digestion altogether. If a chick is found to be dehydrated, the

administration of Darrow's solution and the return to fish formula can be used in order to correct this.

8. FEEDING PROCEDURE

The fish formula is administered using rubber gavage tube and a syringe (Figure 16). Different sizes of gavage tubes are necessary depending on the size of the chick (Table 4). If the tube is too long for the bird (especially for the newly hatched chicks), it must be reduced by doing a diagonal cut and then briefly burning the bevel to remove any sharp/abrasive edges that could harm birds and ensure that the bevel is smooth (Figure 17).

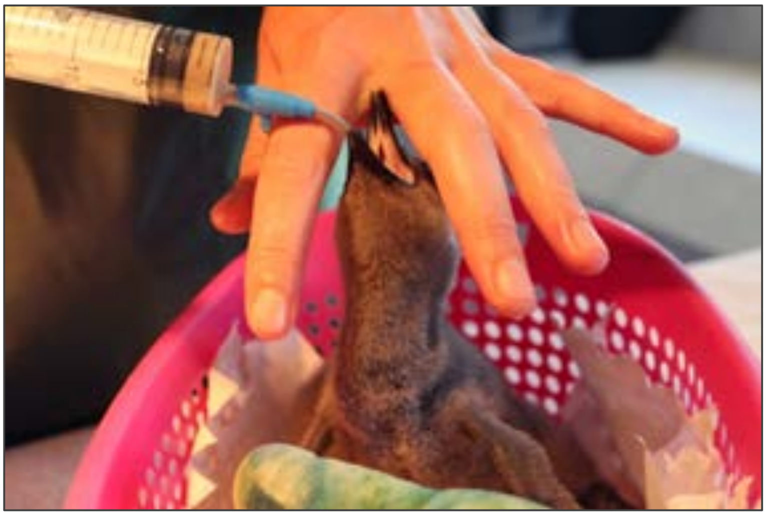

Figure 16. Chick being fed using rubber gavage tube and syringe.

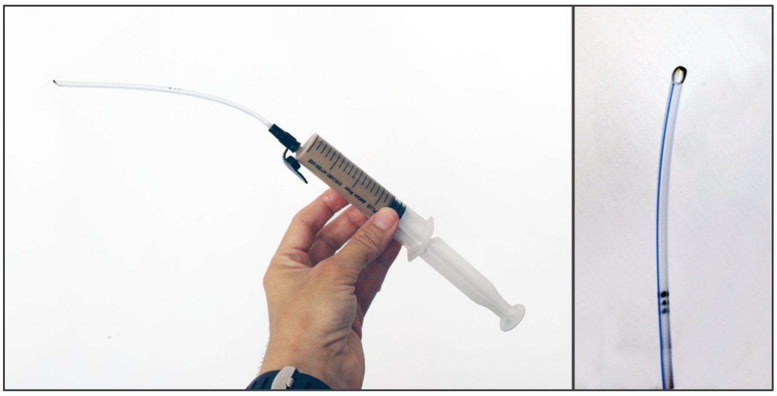

Figure 17. Gavage tube reduced by doing a diagonal cut and then briefly burning the bevel to remove any sharp/abrasive edges to get a smooth bevel.

To minimise handling time, all items that will be used in each feed should be cleaned and prepared beforehand. When feeding chicks under 500 g (chick incubator or brooder crate), the following items must be prepared (Figure 18): a bowl of warm water for cleaning (beak and cloaca), a bowl of water for dipping fish, and a bowl with betadine 10% to clean the umbilicus, cotton gauze to clean/wipe chick, clean and lubricated gavage rubber tubes (AquaMarine® oil or cod liver oil), clean paper towels, a clean plastic basket. To prevent the spread of pathogens, each syringe and gavage rubber tube is labelled with the chick's bird number and is consistently used for the same bird.

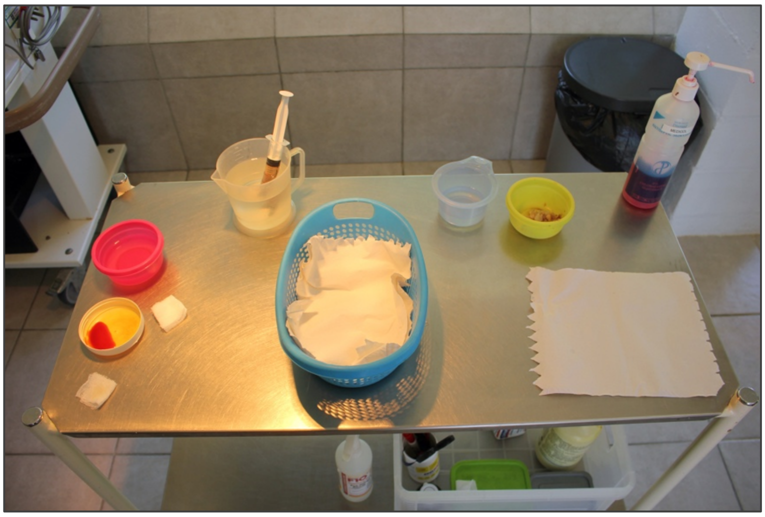

Figure 18. Items prepared beforehand for feeding chicks.

Table 4. Tube size according to chick's age/weight.

Chick age/weight	Tube size
Hatchling (first 3 days)	6FR
Hatchling – 100 g	8FR
100 g – 300 g	10FR
300 g – 500 g	12FR
>500 g	16FR

At each meal, the youngest and healthiest chicks are fed first. Each chick is taken from the incubator or brooder crate and placed on a plastic basket with a clean layer of paper towel ("feeding basket"). The person feeding the chick will make sure that the temperature of the fish formula is adequate (approximately 37-39 °C), and will check that

the gavage rubber tube is appropriately lubricated. The formula in the syringe is shaken to distribute the heat, and then all air within the syringe is taken out before tubing. Before tubing, the stomach is palpated to ensure the chick has digested the fish from its previous meal (fish fillets are not given if the abdomen is full or bloated).

A feeding response is elicited right before feed by forming a “V” with the middle and index fingers on the top of a chick's beak then wiggling the fingers (Figure 19a). The chick should respond by pushing its beak up into the crook of the fingers, and then the gavage tube is introduced a few centimetres into the oesophagus of the bird and the fish formula is slowly injected. If the chick does not display an appropriate feeding response, the beak is gently opened using the thumb and index fingers (Figure 19b). Before injecting the fish formula, it is important to make sure that the gavage tube was properly introduced in the oesophagus (not the trachea) and that the trachea is visible and unobstructed. While injecting the fish formula, it is necessary to observe the mouth of the bird so that the injection can be interrupted in case there is a reflux of fish formula. Before taking the tube out, the gavage tube must be pinched in order to avoid the formula to leak while the tube is being pulled out (which could result in fish formula dripping into the trachea).

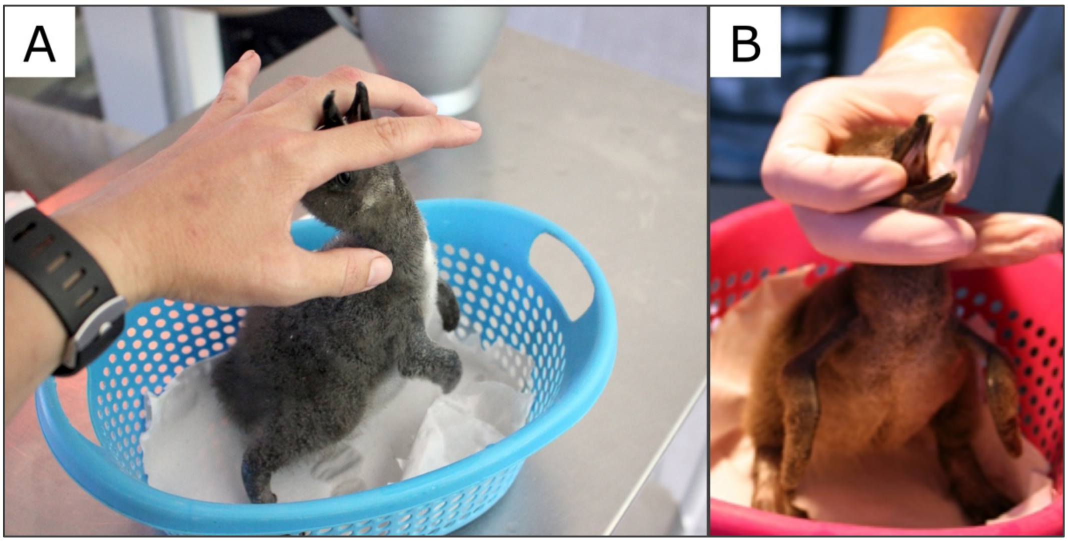

Figure 19. Feeding of small chicks. Legend: (A) chick presenting excellent feeding response; (B) technique for opening a beak when a chick does not display appropriate feeding response.

After the formula was given, fish fillets or fish tails may also be given depending on the feeding regimen. Each fish fillet or fish tail must be dipped into freshwater before it is given to the chick. After each meal, the chick needs to be cleaned (when handling a chick

after feeding, always keep it up right to avoid regurgitation). A cotton gauze with warm water is used to clean the chick's beak. Another piece of gauze with warm water is used to clean around the cloaca and the cloaca itself (Figure 20). Finally, another piece of gauze with 10% betadine is used to clean the umbilical area. After the chick has been cleaned, the soft paper towels and towels placed on the bottom of the pots/crates are changed and the bird is returned to its towel. The paper towel on the feeding basket is then replaced (or, if it has been soiled during feeding, a new basket is used to prevent transferal of bacteria from chick to chick), the person feeding the chicks proceeds to disinfect their hands with Medicol®, and then proceed to feed the next chick.

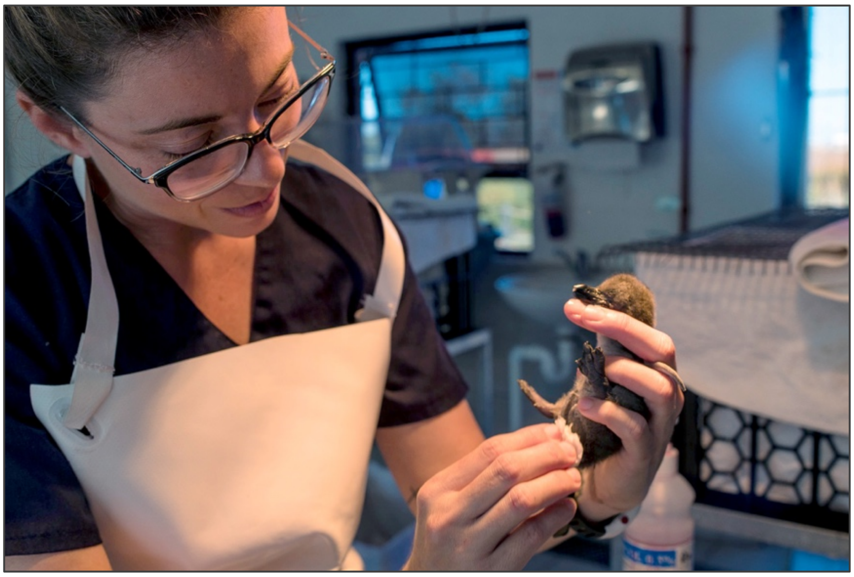

Figure 20. A piece of gauze is used to clean the cloaca after feeding (note that the chick is kept upright during the cleaning to avoid regurgitation). Photo: Jake Lamons.

For chicks exhibiting passive behaviour and lack of initiative to feed, a strategy that can be used is to place another chick of the same age (only if both individuals are healthy) or a toy plush in the feeding basket, to elicit a competitive behaviour.

The procedure to feed chicks over 500 g is similar to that of small chicks, but simpler. Depending on chick's weight/size, they can be fed also using a feeding basket or simply restraining the chicks between the legs of the person feeding, who seats on a low chair or a modified seat (Figure 21). Towels can be used to hold the birds. After feeding, the cleaning procedure is also simplified, and only the chick's beak and face are wiped using a wet cloth in a motion from the base to the tip of the beak. The cloaca and umbilical region are not

routinely cleaned unless they are excessively soiled. It should be noted that whole fishes should always be offered head-first to the bird and if a bird is hesitating to swallow, never pull the fish out of the bird's beak. When offering fish, one should wait until the bird has finished swallowing and appears to be ready for the next fish.

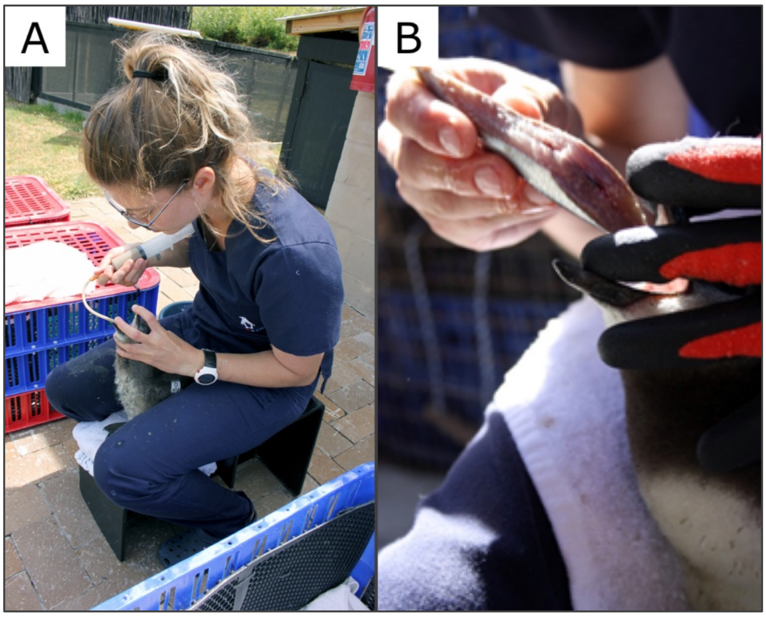

Figure 21. Feeding of larger chicks. Legend: (A) restraining a chick between the legs to administer fish formula; (B) opening the beak to give fish tails.

9. PRE-RELEASE CONDITIONING

The goal of pre-release conditioning is to allow penguin chicks to regain normal physiological and behavioural function in anticipation for release into the wild. In particular, the aim is to ensure that the penguins are waterproof, in good body condition, have appropriate exercise tolerance, and have the ability to perform the full range of behaviors required for survival in the wild.

During pre-release conditioning the penguins are made to swim three times per day (distributed at approximately regular intervals from 08:00h to 17:00h). Each bird is assigned to one of three groups: (a) free swimming, where birds are placed in a pool but allowed to exit at any time, (b) 20-min swimming, where birds are placed in a pool and the exit is only opened after 20 minutes, or (c) 1-hour swimming, where birds are placed in a pool and the

exit is only opened after one hour. Chicks are initially assigned to the free swimming group, and progress to the other stages depending on both clinical condition and the weekly feather grading. Feather grading is conducted by force-swimming the birds for 10-20 min (except if a penguin becomes cold, very distressed or too wet, in which case it is immediately removed), then lifting their feathers to examine if the down is wet (Figure 22).

After a penguin has been promoted to the 1-hour swimming group and its plumage has been found to be waterproof during routine feather grading, it is considered a candidate for release (pending approval for the other release criteria). In this case, a pre-release feather grading is conducted by force-swimming the candidate for one hour and then thoroughly checking the waterproofing of its plumage.

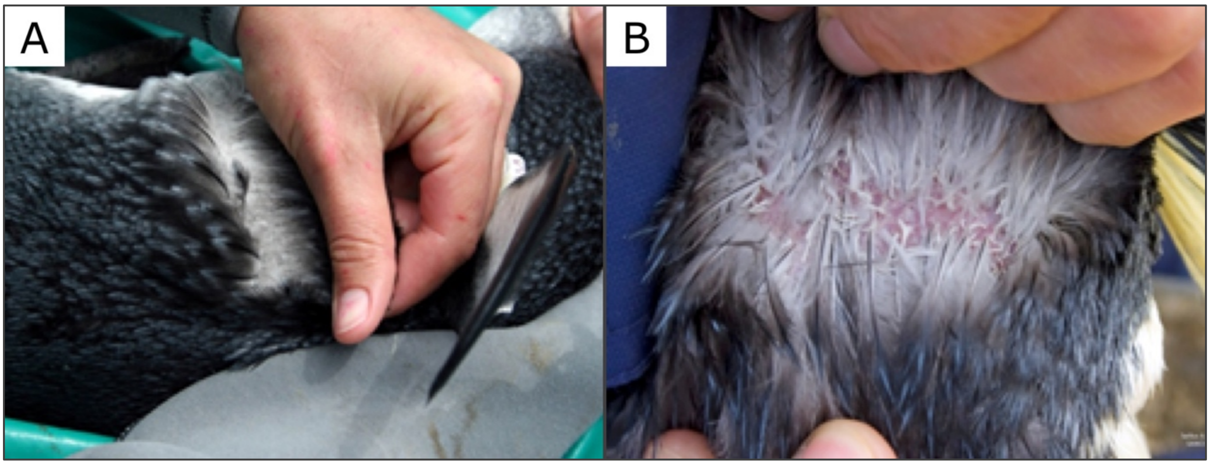

Figure 22. Checking plumage waterproofing. Legend: (A) Lifting feathers to examine the down; (B) Example of an area of the plumage that is not adequately waterproof, as revealed by the fact that the down is wet and the skin is visible.

10. RELEASE CRITERIA

Hand-reared and rehabilitated penguins must be fully recovered and able to meet the athletic demands of survival in the wild prior to release. Current release criteria adopted from SANCCOB are as follows:

- Penguins must be completely waterproof after heavy misting of the entire body or extended periods of time in a pool;

- Good weight and body condition. Penguins' weights should be within 10% of the normal for the species, with consideration given to size, age class, and time of year;
- Normal behaviour (feeding, swimming and interacting appropriately with conspecifics, wariness of humans);
- Birds should have normal physical exams, including hematologic values within normal ranges (packed cell volume 38-48%, total protein 4-6 g/dl), absence of *Plasmodium* sp. and *Borrelia* sp. infections and absence or low parasitemia (<1%) for *Babesia* sp.;
- Transponder inserted into inguinal fold to ensure post-release monitoring.

11. HYGIENE

Strict hygiene protocols must be put into place at the chick-rearing facilities (both the Chick Rearing Unit and the Nursery Unit) to ensure the health and survival of the eggs and chicks. This is achieved by ensuring all equipment and infrastructure is maintained at a high and constant level of hygiene. Aiming to prevent transfer of pathogen from rehabilitation birds or sick birds to the chicks, the staff on duty at the chick-rearing facilities are forbidden from touching/handling other seabirds at the rehabilitation centre, and separate sets of equipment (washing machine, protective gear, clothing, boxes, towels, disinfection equipment, syringes, gavage tubes, etc.) are used for these facilities. Furthermore, access to the chick-rearing facilities is strictly restricted, especially to staff members performing post-mortem examinations or handling birds in Intensive Care Unit. When the veterinary staff has to evaluate chicks at the CRU, this is preferably done early in the morning before they attend to any other birds at the rehabilitation centre.

11.1. Foothbath

Footbaths must be used when access the Chick Rearing Unit and the Nursery Unit. It must have mats to remove the organic material from feet and also be full enough with disinfectant to cover them. The footbaths must be thoroughly washed and filled up at least once a day with benzalkonium-detergent disinfectant (F10SCXD®).

11.2. Personal hygiene

Daily-washed uniforms and shoes are worn by staff working at the chick-rearing facilities. Hands are washed thoroughly with a chlorhexidine disinfectant (Bioscrub®) when arriving at the facility, before and after food preparation, before and after feeding chicks, before and after handling eggs, and when hands are soiled during feeds. Hand washing should follow the instructions of the World Health Organization, taking at least 30 seconds (Figure 23). After hand washing, a chlorhexidine-ethanol disinfectant (Medicol®) must be scrubbed on hands and allowed to air dry before handling eggs and birds from different crates/brooders/pots.

Waterproof aprons must be worn: 1) before feeding chicks from incubators and brooders, preventing bacterial transferral from the older chicks (in crates) to younger ones; 2) when checking Nursery birds, using each apron accordingly to their pen ("healthy", "intermediate" and "sick"), preventing bacterial transferral from the sick chicks to healthy ones.

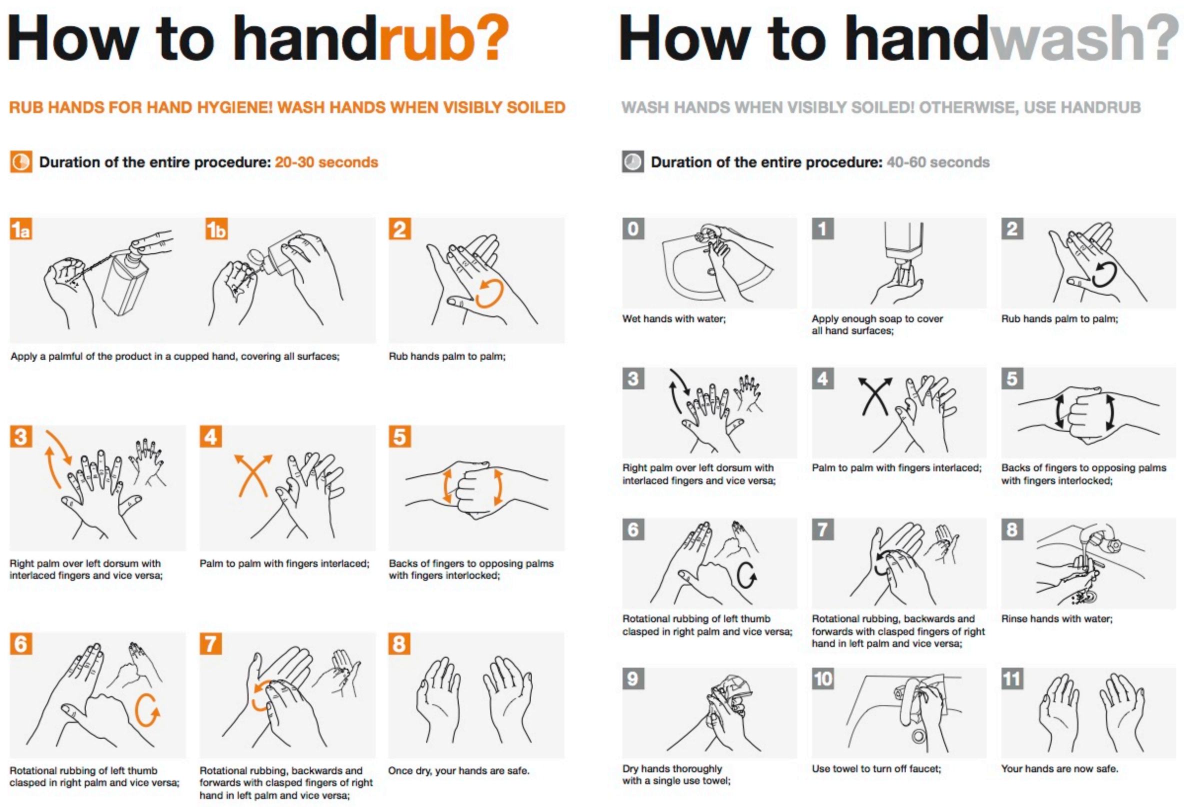

Figure 23. World Health Organisation hand hygiene guidelines in health care.

11.3. Cleaning syringes and tubes

1) Initial rinse: Syringes and tubes used to administer formula to chicks must be rinsed in tap water to remove left-over formula and visible dirt.
2) Step 1: Syringes and tubes are submerged for 10 minutes or more in a bucket with benzalkonium-detergent disinfectant (F10SCXD®). The disinfectant solution must be drawn into the syringes and tubes (so that the product can take effect on their interior), and these must be completely submerged. After 10 minutes, the syringes and tubes are emptied from the disinfectant solution.
3) Step 2: All syringes and tubes are submerged for 20 minutes or more in a bucket with a clean benzalkonium disinfectant (F10SC®). The disinfectant solution must be drawn into the syringes and tubes (so that the product can take effect on their interior), and these must be completely submerged. After 20 minutes, the syringes and tubes are emptied from the disinfectant solution.
4) Final rinse (performed only at Chick Rearing and Nursery units): Syringes and tubes are rinsed with tap water to remove disinfectant solutions, and then allowed to dry.

11.4. General cleaning

Pedal rubbish bins must be used to prevent contamination onto the hands. Daily cleaning routine is performed with benzalkonium disinfectants (F10SC® and F10SCXD®). Kitchen utensils are washed with a benzalkonium-detergent disinfectant (F10SCXD®). A degreasing solution (F919SC®) is used to clean blender and sieves at every time the formula is prepared. Crates, brooders and pots are cleaned with high pressure washer and disinfected (F10SC® and F10SCXD®), and are replaced with clean ones at every morning. Towels/paper used as substrates are replaced after every feed but they can also be replaced as necessary between scheduled cleanings (e.g. birds presenting diarrhea or regurgitation). Plush toys and towels are laundered on a daily basis with laundry powder and chlorine.

All facilities (including the floors, walls, windows, shade nettings, doors, shelves, heat lamps, cabinets, etc.) and all its equipment is thoroughly cleaned and sanitized with chlorine (Medisure®) on a weekly basis, which is also done before fumigation. Once a week, utensils are soaked for 15 minutes in a degreasing solution (F919SC®) to remove any biofilm that

could be present. The inside of fridges is cleaned with benzalkonium disinfectant (F10SC®) on a weekly basis (or more frequently if necessary). Air conditioners are cleaned with chorine (Medisure®) on a weekly basis, and their filters are changed twice a year.

Human incubators are thoroughly cleaned (from the cleanest to dirtiest) twice a week with chlorine (Medisure®) then fumigated for 30 minutes with a benzalkonium-saline solution (F10SC® with a 4% dilution in saline solution). When egg incubators are not in use they must be thoroughly cleaned with chorine (Medisure®), including its trays and rollers. When cleaning is finished, they are fumigated for 30 minutes with a benzalkonium-saline solution (F10SC® with a 4% dilution in saline solution).

All facilities are fumigated twice a year, before and after chick season (which usually starts in November and finishes on February). Incubator air filters are also changed twice every year (before and after every chick season).

12. GENERAL VETERINARY CARE

Incubation and hand-rearing of penguin chicks must be conducted under the supervision of veterinarians.

In addition to providing general supervision to the husbandry and care of the birds, implementing preventative medicine measures, providing care to any birds showing signs of injuries or disease, it is also the role of the veterinary staff to conduct routine veterinary checks in order to detect birds that have on-going health problems that would otherwise go unnoticed. Veterinary checks are conducted on a weekly basis, and comprise:

- Visual inspection (checking for dyspnoea, external lesions, deformities, signs of pain, feather-loss, pox lesions, etc.);
- Pulmonary/air sacs auscultation;
- Hydration status;
- Oral mucosa colour;
- Pododermatitis (bumblefoot) absence/presence and level;
- Plumage stage at that moment (P0 to Blue);
- Case progression assessment and treatments updated if necessary;
- Blood collection.

Blood (approximately 0.1 mL) is collected from the metatarsal vein and used to fill a heparinized capillary tube and prepare a thin blood smear. The capillary tube is centrifuged at 10,000 G for 5 min. The haematocrit (packed cell volume) is measured by using a microhematocrit reader, and the total plasma protein concentration is estimated with a temperature-compensated clinical refractometer. Thin blood smears are fixed and stained with a modified Wright-Giemsa stain (KyroQuick®, Kyro Laboratories) and examined to detect blood parasites and to quantify erythrocytic regeneration and leukocytes.

Faecal samples were collected and fresh smears were examined whenever a penguin presented loss of appetite, slow weight gain or other signs of gastrointestinal problems, aiming to quantify the relative contribution of different bacterial groups (Gram staining and shape). Other diagnostic exams frequently employed to investigate clinical cases include complete cell counts, serum chemistry, radiography, endoscopy, biopsies, among others.

Diseases of primary concern during the hand-rearing of African penguin chicks at SANCCOB include aspergillosis (*Aspergillus* spp.), avian malaria (*Plasmodium* spp.), avian pox (*Avipoxvirus*), babesiosis (*Babesia peircei*), candidiasis (*Candida albicans*), cryptococcosis (*Cryptococcus* sp.), feather-loss disorder (aetiology unknown), high pathogenicity avian influenza (*Influenza A Virus*), limb deformities (nutritional deficiency or excessive weight), relapsing fever (*Borrelia* sp.), pododermatitis, respiratory herpesvirosis (unidentified herpesvirus), helminthiasis (*Contracaecum* spp., *Cardiocephaloides physalis*, *Cyathostoma phenisci*, *Renicola sloanei*, *Tetrabothrius* spp.), and opportunistic bacterial respiratory and gastrointestinal infections.

13. DETAILS ON THE COMMERCIAL PRODUCTS

Tables 5 and 6 provides details on the manufacturers and the composition of the commercial products mentioned in this document.

Table 5. Details of the nutritional supplements and probiotics.

Product	Manufacturer	Composition
AquaMarine® 2 in 1	Vitabiotics	Per mL: Omega-3 fish oil 0.45g, Cod liver oil 0.45g, vitamin A (667IU) 20 µg RE, vitamin D (as D3 400IU) 1 µg , vitamin E 1.1 mg α –TE
Brewer's yeast	Portfolio Pharmaceuticals	Yeasts from *Saccharomyces cerevisiae*
Cani-cal®	Kyron Laboratories	Per g: vitamin A 400 IU, vitamin D3 30 IU, vitamin E 1 IU, iron 4 mg, zinc 1 mg, calcium 70 mg, phosphorus 58 mg
Darrow's solution	Kyron Laboratories	Per mL: potassium chloride 1.5 mg, sodium chloride 2 mg, sodium citrate 2.5 mg, glucose 25 mg
DigestEAZE®	Kyron Laboratories	Per g: amylase 5.000 IU, protease 5.000 IU, lipase 650 IU
Edelweiss® Large Fish Eating Bird Formula		Per capsule: ascorbic acid 47 µg, biotin 28 µg, Ca D pantothenate 6.7 mg, cholcalciferol 2.000 IU, folic acid 160 µg, nicotinic acid 5.7 mg, pyridoxine HCl 1.5 mg, riboflavin 1.2 mg, vitamin A 10.000 IU, vitamin B1 50 mg, vitamin B12 1.5 µg, vitamin E 160 IU, vitamin K 0.5 mg
Karbadust®	Efekto	Carbaryl 50 g/kg
Portfolio® Multivitamin orange	Portfolio Pharmaceuticals	Per tablet: vitamin A 500 IU, vitamin B1 0.5 mg, vitamin C 2.5 mg, vitamin D 25 IU, nicotinamide 2.5 mg
Portfolio® Vitamin B complex	Portfolio Pharmaceuticals	Per tablet: vitamin B1 0.5 mg, vitamin B2 0.25 mg, vitamin B6 0.0625 mg, calcium pantothenate 0.625 mg, nicotinamide 2.5 mg, Brewer's yeast 10 mg
Protexin® Soluble	Kyron Laboratories	Per 10 g/L contains total viable count equal to or greater than 2 × 10^8 cfu/g of the following organisms: *Lactobacillus plantarum, Lactobacillus debrueckii bulgaricus, Lactobacillus acidophilus, Lactobacillus rhamnosus, Bifidobacterium bifidum, Streptococcus salvarius thermophilus*, and *Enterococcus faecium*

Table 6. Details of the disinfectants and cleaning products.

Product	Manufacturer	Composition
BioScrub®	GHS Direct	4% chlorhexidine gluconate, non-ionic surfactants, pH 9-10
F10SC®	Health and Hygiene Ltd	1-10% benzalkonium chloride, 0.1-1% polymeric biguanide hydrochloride; used in 10% dilution in tap water
F10SCXD®	Health and Hygiene Ltd	1-10% benzalkonium chloride, 0.1-1% polymeric biguanide hydrochloride, detergent; used in 10% dilution in tap water
F919SC®	Health and Hygiene Ltd	Biofilm remover and heavy duty degreaser (sodium and silicate compounds 10 to 30%)
Medicol®	Acu-Sol	0.5% chlorhexidine gluconate, 70% ethyl alcohol
Medisure®	Medichem Ltd	Organic chlorine (250 ppm), foaming detergents, corrosion inhibitors, water softening agents
